# A dual regulatory role for the arbuscular mycorrhizal master regulator RAM1 in tomato

**DOI:** 10.1093/jxb/erae210

**Published:** 2024-05-10

**Authors:** Tania Ho-Plágaro, María Isabel Tamayo-Navarrete, Sanja Ćavar Zeljković, Petr Tarkowski, José Manuel García-Garrido

**Affiliations:** Department of Soil and Plant Microbiology, Estación Experimental del Zaidín (EEZ), CSIC, Calle Profesor Albareda no. 1, 18008 Granada, Spain; Department of Soil and Plant Microbiology, Estación Experimental del Zaidín (EEZ), CSIC, Calle Profesor Albareda no. 1, 18008 Granada, Spain; Czech Advanced Technology and Research Institute, Palacky University, Šlechtitelů 27, 78371 Olomouc, Czech Republic; Centre of the Region Haná for Biotechnological and Agricultural Research, Department of Genetic Resources for Vegetables, Medicinal and Special Plants, Crop Research Institute, Šlechtitelů 29, 78371 Olomouc, Czech Republic; Czech Advanced Technology and Research Institute, Palacky University, Šlechtitelů 27, 78371 Olomouc, Czech Republic; Centre of the Region Haná for Biotechnological and Agricultural Research, Department of Genetic Resources for Vegetables, Medicinal and Special Plants, Crop Research Institute, Šlechtitelů 29, 78371 Olomouc, Czech Republic; Department of Soil and Plant Microbiology, Estación Experimental del Zaidín (EEZ), CSIC, Calle Profesor Albareda no. 1, 18008 Granada, Spain; CNB-CSIC, Spain

**Keywords:** Arbuscular mycorrhiza, GRAS, RAM1, strigolactones, tomato, transcriptional regulation

## Abstract

The REQUIRED FOR ARBUSCULAR MYCORRHIZATION1 (RAM1) transcription factor from the GRAS family is well known for its role as a master regulator of the arbuscular mycorrhizal (AM) symbiosis in dicotyledonous and monocotyledonous species, being essential in transcriptional reprogramming for the development and functionality of the arbuscules. In tomato, *SlGRAS27* is the putative orthologue of *RAM1* (here named *SlRAM1*), but has not yet been characterized. A reduced colonization of the root and impaired arbuscule formation were observed in *SlRAM1*-silenced plants, confirming the functional conservation of the RAM1 orthologue in tomato. However, unexpectedly, *SlRAM1*-overexpressing (*UBIL:SlRAM1*) plants also showed decreased mycorrhizal colonization. Analysis of non-mycorrhizal *UBIL:SlRAM1* roots revealed an overall regulation of AM-related genes and a reduction of strigolactone biosynthesis. Moreover, external application of the strigolactone analogue GR24^4DO^ almost completely reversed the negative effects of *SlRAM1* overexpression on the frequency of mycorrhization. However, it only partially recovered the pattern of arbuscule distribution observed in control plants. Our results strongly suggest that SlRAM1 has a dual regulatory role during mycorrhization and, in addition to its recognized action as a positive regulator of arbuscule development, it is also involved in different mechanisms for the negative regulation of mycorrhization, including the repression of strigolactone biosynthesis.

## Teaser

This is the first time a dual role (positive and negative) for RAM1 during mycorrhization has been demonstrated, prompting further exploration into the role of RAM1 across different plant species.

## Introduction

More than 80% of terrestrial plant species form a mutualistic symbiotic association with arbuscular mycorrhizal (AM) fungi. AM formation enhances host plant water and mineral nutrient uptake, and provides plant resilience toward environmental stress including protection against pathogens (Bucher, 2007; Wipf *et al.*, 2019). These positive effects situate AM as a relevant natural resource in sustainable agricultural practices and puts it in the focus of directed scientific approaches leading to increased AM efficiency and responsiveness in plant species of agrobiotecnological interest. Only a thorough understanding of the AM regulatory network and underlying regulatory processes and essential target genes will allow this challenge to be achieved.

Mutual benefit on mycorrhizal plants and AM fungi requires the formation of arbuscules in the host root cells, which are specialized intraradical and highly branched fungal structures (Gutjahr and Parniske, 2013). Signal perception by the symbionts, transcriptional reprogramming in the host plant, as well as hormone signalling have been proposed as molecular mechanisms underlying the adaptive changes in roots leading to the formation and function of the arbuscules (Pimprikar and Gutjahr, 2018). Thus, DELLA is a GRAS protein that negatively regulates gibberellin (GA) signalling (Davière and Achard, 2013), and the DELLA–GA module plays a central role in regulating arbuscule formation (Floss *et al.*, 2013; Martin-Rodriguez *et al.*, 2016). Interestingly, it has been shown that other transcription factors (TFs) and transcriptional regulators (TRs) also belonging to the GRAS [for GA_3_ INSENSITIVE (GAI), REPRESSOR OF GAI (RGA), and SCARECROW (SCR)] family play a pivotal role in the transcriptional regulatory network that controls AM development (Ho Plágaro and García-Garrido, 2022).

Among GRAS TFs involved in AM symbiosis, RAM1 (REQUIRED FOR ARBUSCULAR MYCORRHIZATION 1) has received the most attention. RAM1 was initially described in *Medicago truncatula* (Gobbato *et al.*, 2012), and *RAM1* gene orthologues with similar functions were characterized in *Lotus japonicus* (Xue *et al.*, 2015), *Petunia hybrida* (Rich *et al*., 2015), and, most recently, in the monocot species *Brachypodium distachyon* (Müller *et al.*, 2020). *RAM1* expression is regulated by CYCLOPS, a symbiotic TF of the common SYM pathway activated in the root after rhizobia and AM fungi recognition. CYCLOPS participates with DELLA in the regulatory complex that activates *RAM1* transcription (Pimprikar *et al*., 2016).

As has been shown in studies with *ram1* mutants, RAM1 is a major regulator of AM symbiosis formation. *Ram1* mutant plants displayed defective symbiosis with limited arbuscule branching and reduced hyphal colonization of the root (Gobbato *et al.*, 2012; Park *et al.*, 2015; Rich *et al*., 2015; Xue *et al.*, 2015; Pimprikar *et al*., 2016). Gene expression analysis and RNA-seq experiments with *ram1* mutants and roots overexpressing *RAM1* (Park *et al.*, 2015; Luginbuehl *et al*., 2017; Rich *et al*., 2017) have revealed that RAM1 directly or indirectly participates in the regulation of AM-related gene expression. For example, it has been shown that RAM1 regulates TF genes such as the GRAS *RAD1* (REQUIRED FOR ARBUSCULE DEVELOPMENT1) (Park *et al.*, 2015), some AP2-domain TF genes of the WRINKLED5 (WRI5) family in *M. truncatula* (Jiang *et al.*, 2018), *CBX1* in *L. japonicus* (Xue *et al.*, 2018), and also genes involved in arbuscule development and periarbuscular membrane nutrient exchanges, including *FatM*, *RAM2*, *STR*, and *PT4* (Gobbato *et al.*, 2012; Park *et al.*, 2015; Pimprikar *et al.*, 2016; Luginbuehl *et al*., 2017).

Several observations point to the hypothesis that variations in the biological roles of TF/TRs denote diversification features that can be attributed to diversity in AM symbiosis regulatory networks among mycorrhizal host species. Thus, several predicted RAM1 target genes were differently regulated between *L. japonicus*, *M. truncatula*, and *P. hybrida* (Park *et al.*, 2015; Pimprikar *et al*., 2016; Luginbuehl *et al*., 2017; Rich *et al*., 2017), suggesting that slight differences in regulation of AM-related genes even exist between relatively closely related plant species (Pimprikar and Gutjahr, 2018). Furthermore, RAD1 protein, a GRAS TF which interacts with RAM1, is essential for arbuscule development in *L. japonicus* (Xue *et al.*, 2015), while the *rad1* mutant develops normal arbuscule branching in *M. truncatula* although with reduced colonization levels (Park *et al.*, 2015). In this scenario, it is tempting to speculate that some functional diversification of GRAS TF/TR functions in AM symbiosis between plant species has occurred during evolution.

Among the AM-induced tomato GRAS TFs, Ho-Plágaro *et al.* (2019) identified putative orthologues to genes with a previously established functionality in AM regulation in other plant species, including the clades NSP2, RAM1, RAD1, LISCL, and the AM-host exclusive clade SCLB, which includes the MIG1 transcription factor (Maillet *et al.*, 2011; Gobbato *et al.*, 2012; Yu *et al.*, 2014; Fiorilli *et al.*, 2015; Xue *et al.*, 2015; Heck *et al.*, 2016; Rich *et al*., 2017; Ho-Plágaro *et al.*, 2019). *SlGRAS27* (*SlRAM1*), which is also a GRAS gene induced upon mycorrhization and expressed in arbuscule-containing cells, was characterized as the putative homologue of *MtRAM1* and *PhATA/RAM1* from *Medicago* and *Petunia*, respectively (Ho-Plágaro *et al.*, 2019). Here we report a functional analysis of the *SlRAM1* gene using RNAi and overexpressing (OE) hairy root composite tomato plants. The results obtained suggest that *SlRAM1* has a dual regulatory role during mycorrhization and, in addition to its accepted role as a positive regulator of arbuscule development, the SlRAM1 TF is also involved in different mechanisms for the negative regulation of mycorrhization.

## Materials and methods

### Plant growth and AM inoculation

*Solanum lycopersicum* seeds were surface sterilized with a 5 min soaking using 2.35% w/v sodium hypochlorite, subjected to shaking at room temperature for 1 d in the dark, and germinated on a sterilized moistened filter paper for 4 d at 25 °C in the dark. Germinated seeds were placed on vermiculite for hypocotyl elongation for 1 week. Each seedling was transferred to a 500 ml pot containing an autoclave-sterilized (20 min at 120 °C) mixture of expanded clay, washed vermiculite, and coconut fibre (2:2:1, v/v/v). In the AM-inoculated treatments, the plants were inoculated with a piece of monoxenic culture in Gel-Gro medium produced according to Chabot *et al.* (1992), containing 50 spores of *Rhizophagus irregularis* (DAOM 197198) and infected carrot roots. Plant growth took place in a growth chamber (day:night cycle, 16 h:8 h, 24 °C:20 °C; relative humidity 50%). One week after planting and weekly thereafter, the pots were given 20 ml of a modified Long Ashton nutrient solution (Hewitt, 1966) containing 25% (325 µM) of the standard phosphorus concentration (1.3 mM) to prevent mycorrhizal inhibition as a result of excess phosphorus. In the case of non-mycorrhizal plants, the same modified Long Ashton solution was used. Plants were harvested at different times after inoculation. The root system was washed and rinsed several times with tap water and used for the different measurements according to the nature of the experiments.

For the mycorrhizal experiment using the *Funneliformis mosseae* AM fungus, *F. mosseae* (Schüßler and Walker, 2010) inoculum was obtained from the EEZ germplasm collection. The fungus was propagated using sorghum (*Sorghum bicolor*) as the host, and the infected roots, hyphae, spores, and substrates were collected and used as inoculum (40 g per pot).

For the phosphate- (P) starvation experiment, a mixture of washed vermiculite and sand (1:1, v/v) in 500 ml pots was used. Uninoculated plants were watered with 20 ml of complete Long Ashton nutrient solution (Hewitt, 1966) three times a week. After 2 weeks, the substrate was washed with 1 litre of tap water before the application of the P-deficiency treatment. For the following 2 weeks, plants were watered every day with 25 ml of Long Ashton solution at the standard phosphorus concentration for the control treatment (5.2 mM Pi; +P) or without phosphorus for the P-starvation treatment (0 mM Pi; –P). Afterwards, the substrate was washed with 500 ml of the +P or –P nutrient solution to remove compounds accumulated during plant growth. Plants were kept for 48 h in the growth chamber to allow the production of ‘fresh’ root exudates and irrigated to field capacity with the corresponding nutrient solution after 24 h. After the 48 h period, fresh root exudates from each plant were collected individually, and strigolactones (SLs) were purified as described below. On the same day, roots were collected, weighed, snap-frozen in liquid nitrogen, and kept at –80 °C until analysis.

For the GR24 experiments, a stock solution of 10 mM GR24^4DO^ (StrigoLab, Torino, Italy) dissolved in acetone was added to the Long Ashton nutrient solution (Hewitt, 1966) to reach a 0.05 µM final concentration, and 25 ml were applied to *R. irregularis*-inoculated plants twice a week. Control plants for these experiments were fertilized with the Long Ashton nutrient solution supplemented with the respective amount of acetone.

### Staining of AM fungus and estimation of root colonization

The non-vital trypan blue histochemical staining procedure was performed according to Phillips and Hayman (1970). Stained roots were observed using a light microscope, and the intensity of root cortex colonization by AM fungus was assessed as described by Trouvelot (1986) using the Mycocalc software (http://www2.dijon.inra.fr/mychintec/Mycocalc-prg/download.html). Several parameters were measured, including the frequency of colonization (%F); the intensity of colonization in the entire root system (%M=relative mycorrhizal intensity); intensity of the mycorrhizal colonization in the root fragments (%m=absolute mycorrhizal intensity); the arbuscular abundance along the entire length of the root (%A=relative arbuscule richness), and the arbuscular abundance in mycorrhizal parts of root fragments (%a=absolute arbuscule richness). At least five microscope slides were analysed per biological replicate, and each slide contained 30 root pieces of 1 cm. Alternatively, 120 root intersects from each of these slides were analysed by the magnified intersections method according to McGonigle *et al.* (1990) to determine the relative abundance of different AMF structures, including vesicles, total arbuscules, and arbuscules from three morphological categories. As previously described (Ho-Plágaro *et al*., 2021), three arbuscule typologies were defined as follows: class a, small arbuscules with no fine branches; class b, arbuscules with intermediate intensity of trypan blue stain occupying almost all of the plant cell; and class c, arbuscules with a high intensity of trypan blue stain occupying the whole plant cell.

For a detailed imaging of arbuscule morphology, roots were incubated in 50% ethanol for 4 h and in 20% KOH for 3 d. Then, roots were washed with dH_2_O, incubated on 0.1 M HCl for 2 h, washed again with dH_2_O and 1× phosphate-buffered saline (PBS ), dark-incubated overnight in 10 μg ml^–1^ wheat germ agglutinin (WGA)–Alexa Fluor 488 conjugate (Molecular Probes, Eugene, OR, USA) in 1× PBS, and washed in 1× PBS before its microscopic observation. *Z*-stack images were obtained using a laser scanning confocal fluorescence microscope (C-1; Nikon).

### Plasmid construction and hairy root transformation

The full-length cDNA gene sequence of *SlRAM1* (Solyc02g094340.1, according to the updated ITAG 4.0 annotation) and a 233 bp *SlRAM1* RNAi fragment were amplified from *S. lycopersicum* cDNA of roots infected by the AM fungus *R. irregularis*. Amplifications were carried out by PCR using the iProof High Fidelity DNA-polymerase (BioRad, Hercules, CA, USA) and specific primers (Supplementary Table S1). PCR fragments were introduced in the pENTR/D-TOPO (Invitrogen, Carlsbad, CA, USA) vector and sequenced. pENTR/D-TOPO containing the *SlRAM1* gene and the RNAi *SlRAM1* fragment were subsequently recombined into pUBIcGFP-DR (Kryvoruchko *et al.*, 2016) and pK7GWIWG2_II-RedRoot (https://gatewayvectors.vib.be/) vectors, respectively, using the GATEWAY technology (Invitrogen).

For hairy root transformation, *Agrobacterium rhizogenes* MSU440 cultures harbouring the corresponding OE (*UBIL:SlRAM1*) and RNAi constructs were used to transform *S. lycopersicum* cv Moneymaker plantlets according to Ho-Plágaro *et al.* (2018). Composite plants were transferred to pots and followed the same plant growth conditions as explained earlier. Screening and selection of DsRed (transformed) roots was done by observation under a fluorescent stereomicroscope Leica M165F.

### RNA extractions and gene expression quantification

For the quantitative reverse transcription–PCR (RT–qPCR) experiments, representative root samples from each root system were collected, immediately frozen in liquid nitrogen, and stored at −80 °C until RNA extraction. Total RNA was isolated from ~0.2 g samples using the RNeasy Plant Mini Kit (Qiagen) following the manufacturer’s instructions, and treated with RNase-free DNase. A 1 µg aliquot of DNase-treated RNA was reverse transcribed into cDNA using the iScript™ cDNA synthesis kit (BioRad) following the supplier’s protocol. For the qPCR, a 20 μl PCR was prepared containing 1 μl of diluted cDNA (1:10), 10 μl of 2× SYBR Green Supermix (BioRad), and 200 nM of each primer using a 96-well plate. A negative control with the RNA sample before reverse transcription was used to confirm that the samples were free from DNA contaminations. The PCR program consisted of a 3 min incubation at 95 °C, followed by 35 cycles of 30 s at 95 °C, 30 s at 58–63 °C, and 30 s at 72 °C. The specificity of the PCR amplification procedure was checked using a melting curve after the final PCR cycle (70 steps of 30 s, from 60 °C to 95 °C, at a heating rate of 0.5 °C). Experiments were carried out on three biological replicates, and the threshold cycle (Ct) value for each biological replicate was determined from three technical replicates. The relative transcription levels were calculated by using the 2^−ΔΔCt^ method (Livak and Schmittgen, 2001). The Ct values of all genes were normalized to the geometric mean of Ct values from the *LeEF-1a* (accession no. X14449) and actin (NM_001321306.1) housekeeping genes.

The RT–qPCR data for each gene were shown as relative expression with respect to the ‘reference treatment’ to which was assigned an expression value of 1. All genes whose transcript abundance was measured by RT–qPCR and the corresponding primers used are listed in Supplementary Table S1.

### RNA sequencing analysis

Three root pools from control plants transformed with the empty vector and three pools of *UBIL:SlRAM1* composite plants were used. Each pool was composed of a representative mixture of two root systems from two composite plants. Total RNA was extracted using the Rneasy Plant Mini Kit (Qiagen). The quality and quantity of total RNA samples were assessed using a NanoDrop 1000 spectrophotometer (Thermo Scientific) and samples were normalized at the same concentration (6 μg, 300 ng μl^−1^). Later, samples were sent to Sistemas Genómicos SL (Paterna, Valencia, Spain) for cDNA library preparation and sequencing using an Illumina HiSeq1000 machine. Raw RNA-seq data were deposited in the NCBI Short Read Archive (SRA) with accession no. PRJNA523214.

The TopHat v.2.1.0 algorithm (Trapnell *et al.*, 2009) was used to align reads from the RNA-seq experiment to the Tomato Genome Reference Sequence SL3.0 provided by the Sol Genomics consortium at https://solgenomics.net/organism/Solanum_lycopersicum/genome, using the Itag 3.10 annotation. Then, low quality reads were removed from the map through Picard Tools (http://picard.sourceforge.net), and high quality reads were selected for assembly and identification through Bayesian inference using the Cufflinks v.2.2.1 algorithm proposed by Trapnell *et al.* (2010). The gene quantification process was performed by the htseq-count 0.6.1p1 tool (Anders *et al.*, 2015). Isoform quantification and differential expression were carried out through the DESeq2 method (Anders *et al.*, 2015).

For the analysis of transcriptional changes occurring during arbuscular mycorrhization, data from a previous experiment performed by Ho-Plágaro *et al.* (2021) (NCBI Bioproject PRJNA509606) was used and RNA-seq sequence processing was performed as explained above.

### Root exudate collection and purification of strigolactones

Crude root exudates were collected individually by applying 1 litre of tap water to each pot, and vacuum-filtered through glass filters. Then, exudates were concentrated and purified by solid phase extraction through Telos C18 (EC) SPE columns (Octadecyl 500 mg/3 ml) (Telos, Kinesis, UK) using an SPE vaccum manifold (Supelco). For that, SPE columns were first pre-equilibrated with 5 ml of 100% acetone, and washed with 5 ml of H_2_O_d_. Then, 1 litre of each exudate solution was loaded onto the pre-equilibrated columns. Each column was washed with 5 ml of 40% acetone, and the exudates were eluted with 5 ml of 60% acetone and collected in 10 ml amber tubes. Purified root exudates were stored at –80 °C until use.

### Strigolactone analysis by UHPLC

A 15 µl aliquot of 25 nM GR24 (internal standard) was added to 150 µl of purified root exudate, evaporated to dryness under a vacuum at 40 °C, and redissolved in 15 µl of ACN (acetonitrile). Samples were analysed on an Nexera X2 UHPLC (Shimadzu, Handels GmbH, Kyoto, Japan) coupled with an MS-8050 (Shimadzu, Handels GmbH, Kyoto, Japan). Chromatographic separation was performed with an ACQUITY BEH C18 column (50 × 2.1 mm; 1.7 µm; Waters, Milford, MA, USA) with a corresponding pre-column. The column temperature was 40 °C, the flow rate 0.50 ml min^–1^, and the injection volume was 5 µl. The mobile phase consisted of water (A) and methanol (B) with gradient elution as follows: 40% B for 1 min, increasing to 60% B for 3 min, then increasing to 90% B for 1 min, and keeping isocratic for 0.5 min. Initializing conditions were set after 0.1 min, and then equilibrating for 1.9 min. The mass spectra were obtained via electrospray ionization in positive mode with the following operating parameters: capillary voltage –3000 V; interface voltage 4 kV; interface temperature 300 °C; heating and drying gas flow 10 l min^–1^; and nebulizing gas flow 3 l min^–1^. The MS/MS spectra of the protonated molecular ions [M+H]^+^ or sodium adducts [M+Na]^+^ were acquired (Supplementary Table S2). SLs were identified by the comparison of the retention times (Rt) and multiple reaction monitoring (MRM) transitions with those of authentic standards. Software LabSolutions 5.72 (Shimadzu, Handels GmbH, Kyoto, Japan) was used for data processing.

### Statistical analysis

When two means were compared, the data were analysed using a two-tailed Student’s *t*-test. For comparisons among all means, a one-way ANOVA was performed followed by the Tukey’s multiple comparison test. Graphpad Prism v.6.01 (Graphpad Software, San Diego, CA, USA) was used to determine statistical significance. Differences at *P*<0.05 were considered significant.

## Results

### The function of *RAM1* is conserved in its tomato orthologue *SlGRAS27*

**Fig. 1.**
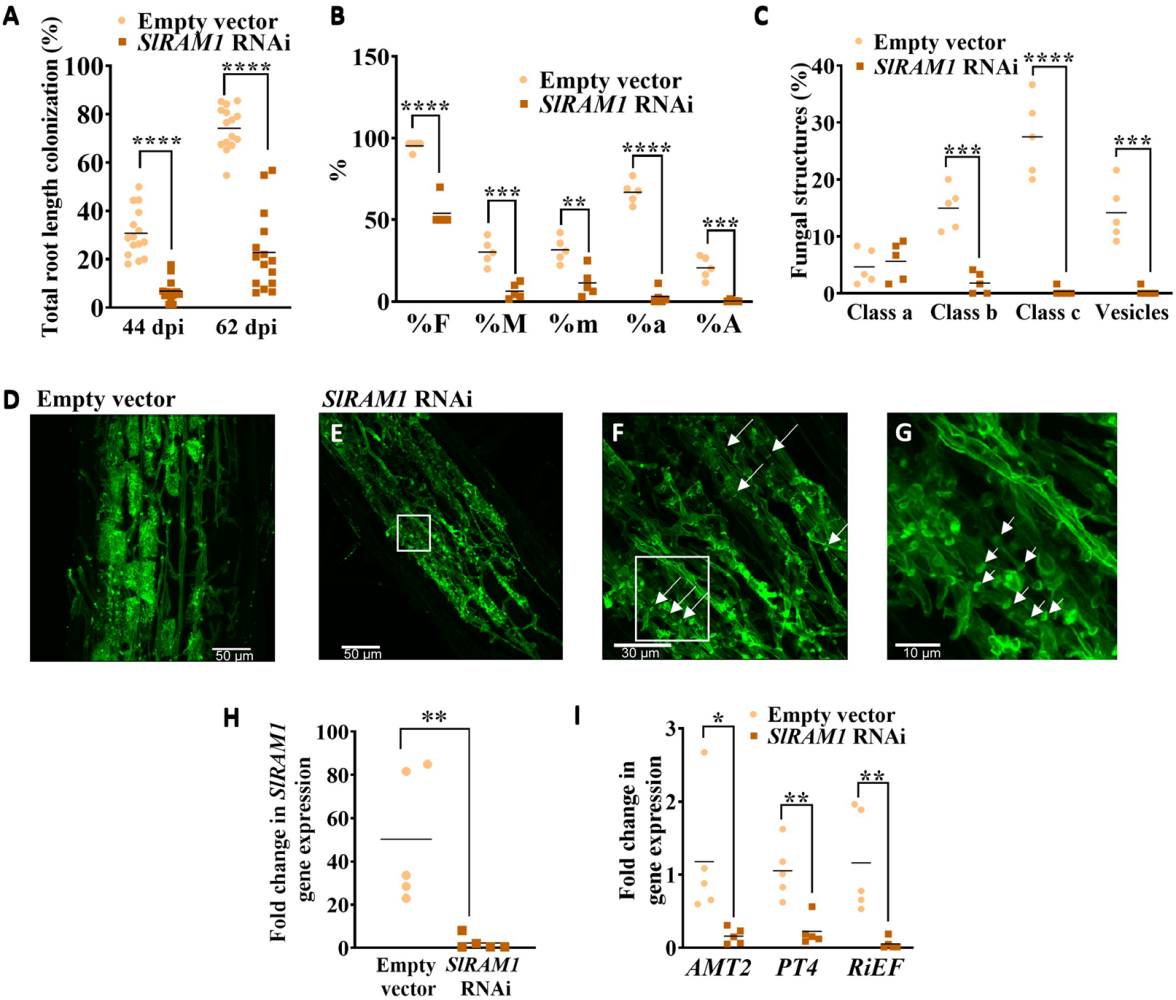
Mycorrhizal phenotype of *SlRAM1* RNAi composite tomato plants. (A) The percentage of total root length colonized was analysed in root systems of *SlRAM1* RNAi and control (transformed with the empty vector) composite tomato plants at 44 and 62 days post-inoculation (dpi) with the arbuscular mycorrhizal fungus *R. irregularis* (*n*=15). (B and C) Some of these samples from 44 dpi (*n*=5) were randomly selected to analyse the mycorrhization parameters (B), as well as the percentage of root intersects with the presence of vesicles and/or with a prevalence of arbuscules from three different morphological types: class a (small and unbranched), class b (middle size), or class c (highly intense occupying the whole plant cell) (C). (D–G) Roots were subjected to WGA–Alexa Fluor 488 staining and visualized by confocal laser scanning microscopy (scale bars=30 μm). Fully developed arbuscules were observed in hairy roots transformed with the empty vector (D), while a defective arbuscule branching and frequent septa in the fungal hyphae (white arrows) appeared in the *SlRAM1* RNAi roots (E–G). (F) is a higher magnification image of (E), and (G) is a higher magnification image of the boxed area in (F). (H, I) Expression of several genes was measured by RT–qPCR in *SlRAM1* RNAi and control roots (*n*=5), including *SlRAM1* to prove its effective silencing in the *SlRAM1* RNAi roots (H), and several arbuscular mycorrhizal marker genes (I). Gene expression data were represented with respect to mycorrhizal control roots (or with respect to *SlRAM1* RNAi roots in the case of *SlRAM1* gene expression quantification), for which the expression level was designated as 1. Significant differences (Student’s *t*-test) between plants transformed with the empty vector and *SlRAM1* RNAi plants are indicated with asterisks (*P<0.05; **P<0.01; ***P<0.001; ****P<0.0001). %F=frequency of colonization; %M=relative mycorrhizal intensity; %m=absolute mycorrhizal intensity; %a=absolute arbuscule richness; %A=relative arbuscule richness; *AMT2*=*Ammonium transporter 2*; *PT4*=*Phosphate transporter 4*; *RiEF*=*R. irregularis Elongation factor.*

In a previous work, we identified *SlGRAS27* (Solyc02g094340.1) as the putative orthologue of *PhATA/RAM1*, based on phylogenetic analysis and its gene induction upon mycorrhization, specifically associated with arbuscule-hosting cells (Ho-Plágaro *et al.*, 2019). To validate that tomato *SlGRAS27* (here named *SlRAM1*) conserves the symbiotic function of *PhATA/RAM1* orthologue genes from other species, we tested the effect of *SlRAM1* silencing in mycorrhizal tomato roots. As expected, a significant clear decrease in the percentage of the root length colonized by the AM fungus *R. irregularis* was observed in the *SlRAM1* RNAi roots at 44 and 62 days post-inoculation (dpi) (Fig. 1A). Randomly selected mycorrhizal roots from the first harvesting time (44 dpi) showed an overall reduction in mycorrhizal parameters and in all arbuscule classes (Fig. 1B, C). Also, a significant decline on the fungal vesicle number was observed upon *SlRAM1* silencing, which is a sign of aberrant arbuscule function, as described for *ram1* mutants in other plant species (Pimprikar *et al*., 2016; Luginbuehl *et al*., 2017). Microscopic examination of WGA–Alexa488 fluorescent-stained hairy roots was performed. While well-developed and highly branched arbuscules were often found in the control roots transformed with the empty vector (Fig. 1D), *SlRAM1* RNAi showed an impaired arbuscule formation, accompanied by the presence of abundant highly septate fungal hyphae in the cortex (Fig. 1E–G), suggesting the presence of fungal stress and degeneration. RT–qPCR analysis showed that *SlRAM1* gene silencing in the *SlRAM1* RNAi roots was effective (Fig. 1H), and was accompanied by a significant decrease in the expression of AM-related genes, including *S. lycopersicum* transporter genes for arbuscule functionality (*PT4* and *AMT2*) and the *R. irregularis RiEF* constitutive gene (Fig. 1I). Overall, the decreased mycorrhization and aberrant arbuscule phenotype, together with a decreased vesicle number in the *SlRAM1* RNAi tomato roots, resemble the phenotype described for the *ram1* mutants in petunia, *Lotus*, and *Medicago*, and thus we conclude that *SlRAM1* from tomato conserves the function of its *PhATA/RAM1* orthologues from different species previously reported as positive master regulators of arbuscule development (Gobbato *et al.*, 2012; Xue *et al.*, 2015; Rich *et al*., 2015).

### SlRAM1 overexpression negatively affects AM symbiosis

**Fig. 2.**
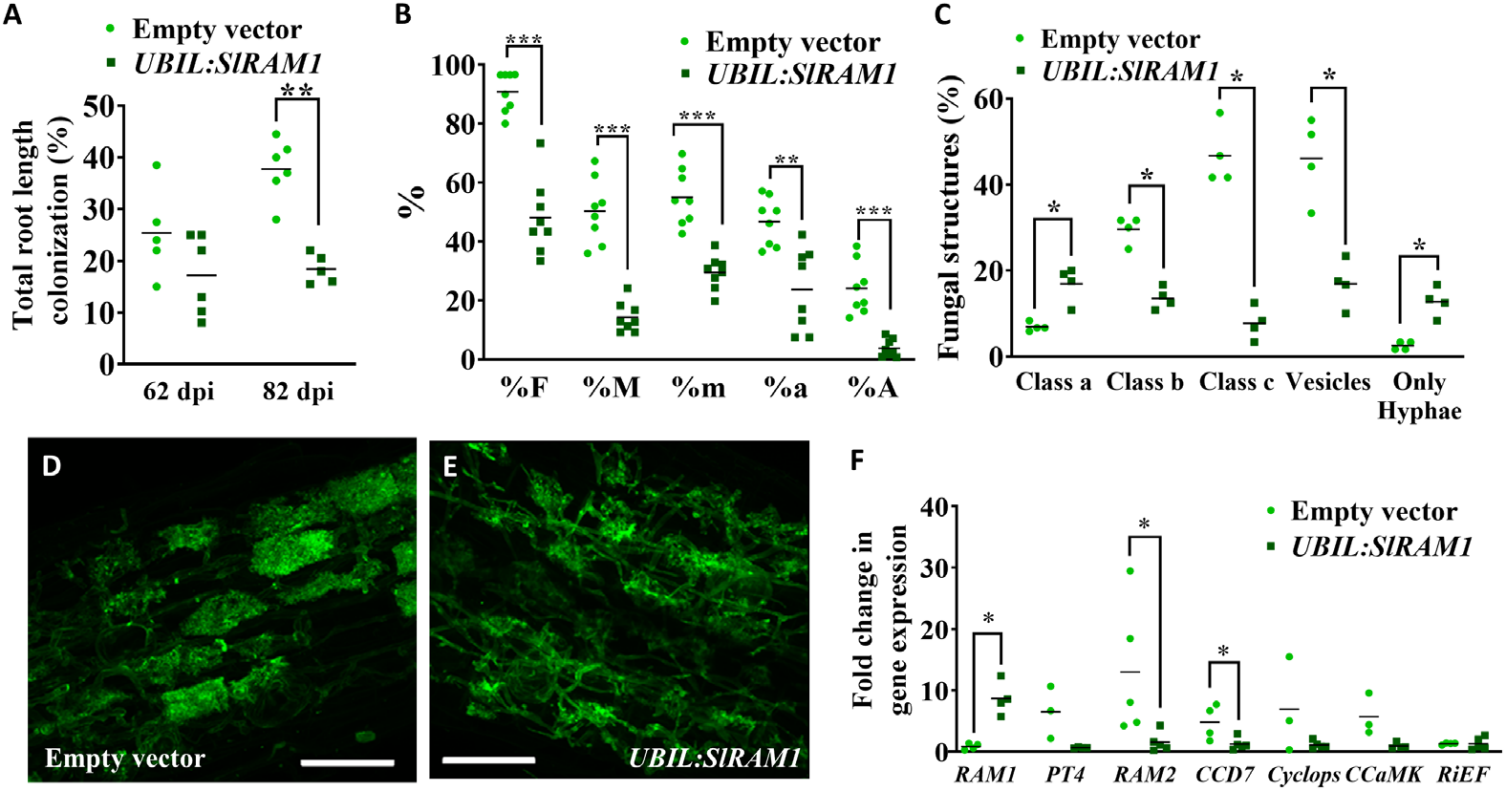
Mycorrhizal phenotype of *SlRAM1-*overexpressing composite tomato plants. (A) The percentage of total root length colonized was analysed in root systems of *UBIL:SlRAM1* and control (transformed with the empty vector) composite tomato plants at 62 and 82 dpi with the arbuscular mycorrhizal fungus *R. irregularis*. Some of these samples from 82 dpi (*n*≥4) were randomly selected to analyse the mycorrhization parameters (B) as well as the percentage of root intersects with the presence of vesicles and/or with a prevalence of small-, middle- or full-sized arbuscules (classes a, b, and c, respectively) (C). (D, E) Roots were subjected to WGA–Alexa Fluor 488 staining and visualized by confocal laser scanning microscopy (scale bar=50 μm). While fully developed arbuscules were easily observed in hairy roots transformed with the empty vector (D), they were difficult to find in *UBIL:SlRAM1* roots (E). (F) Expression of several genes was measured by RT–qPCR in *UBIL:SlRAM1* and control roots including *SlRAM1*, to prove its effective overexpression in the *UBIL:SlRAM1* roots, and several AM marker genes. Gene expression data were represented with respect to mycorrhizal *UBIL:SlRAM1* roots (or with respect to control roots in the case of the *SlRAM1* gene), for which the expression level was designated as 1. Significant differences (Student’s *t*-test) between plants transformed with the empty vector and *UBIL:SlRAM1* plants are indicated with asterisks (*P<0.05; **P<0.01; ***P<0.001) (*n*≥3). %F=frequency of colonization; %M=relative mycorrhizal intensity; %m=absolute mycorrhizal intensity; %a=absolute arbuscule richness; %A=relative arbuscule richness; *PT4*=*Phosphate transporter 4*; *RiEF*=*R. irregularis Elongation factor*; *RAM1*=*Required for Arbuscular Mycorrhization 1*; *RAM2*=*Required for Arbuscular Mycorrhization 2*; *CCD7*=*Carotenoid cleavage dioxygenase 7*; *CCaMK*=*Ca*^*2+/*^*calmodulin (CaM)-dependent protein kinase*; *Cyclops*=*Transcriptional activator Cyclops*.

To obtain further insight into the function of *SlRAM1* in AM symbiosis, we tested the effect of *SlRAM1* overexpression (*UBIL:SlRAM1*) in mycorrhizal tomato roots. Unexpectedly, roots overexpressing *SlRAM1* under the control of the maize ubiquitin (*Ubil*) promoter showed a reduced mycorrhizal root length, that was significant and especially pronounced at the second harvesting time at 82 dpi, where *UBIL:SlRAM1* roots presented a 41% decrease in the root length colonized by the AM fungus *R. irregularis* (Fig. 2A). We decided to perform an in-depth characterization of the phenotype of these *UBIL:SlRAM1* tomato composite plants harvested at 82 dpi. Microscopic analysis revealed that *UBIL:SlRAM1* roots showed a significant decrease in all the mycorrhizal parameters compared with control roots (Fig. 2B). Moreover, *UBIL:SlRAM1* roots showed a significant decrease in the relative content of vesicles, as well as in the percentage of medium- and large-sized arbuscules (classes b and c, respectively) (Fig. 2C). Actually, fully developed arbuscules typically present in control roots (Fig. 2D) were difficult to find in the inspected *UBIL:SlRAM1* roots, even in highly mycorrhized root areas (Fig. 2E). The successful up-regulation of *SlRAM1* gene expression in the *UBIL:SlRAM1* hairy roots was confirmed, and the expression of a subset of AM-related genes was quantified (Fig. 2F). Although expression of the *R. irregularis RiEF* constitutive gene was not altered in the *UBIL:SlRAM1* mycorrhizal hairy roots with respect to the control mycorrhizal roots, repression of the *PT4* and *RAM2* marker genes for arbuscule functionality was found in these roots. Moreover, the *CCD7* gene involved in SL biosynthesis, as well as the *Cyclops* and *CCaMK* genes participating in the common symbiosis signalling pathway, were also repressed in mycorrhizal roots upon *SlRAM1* overexpression (Fig. 2F). To check if the results obtained are reproducible for other AM fungus species, we also analysed the effect of *SlRAM1* overexpression in *F. mosseae*-inoculated roots and, similarly, a decrease in the mycorrhizal root length was observed in *UBIL:SlRAM1* roots (Supplementary Fig. S1).

### Transcriptomic changes triggered by *UBIL:SlRAM1* in AM-related genes

**Fig. 3.**
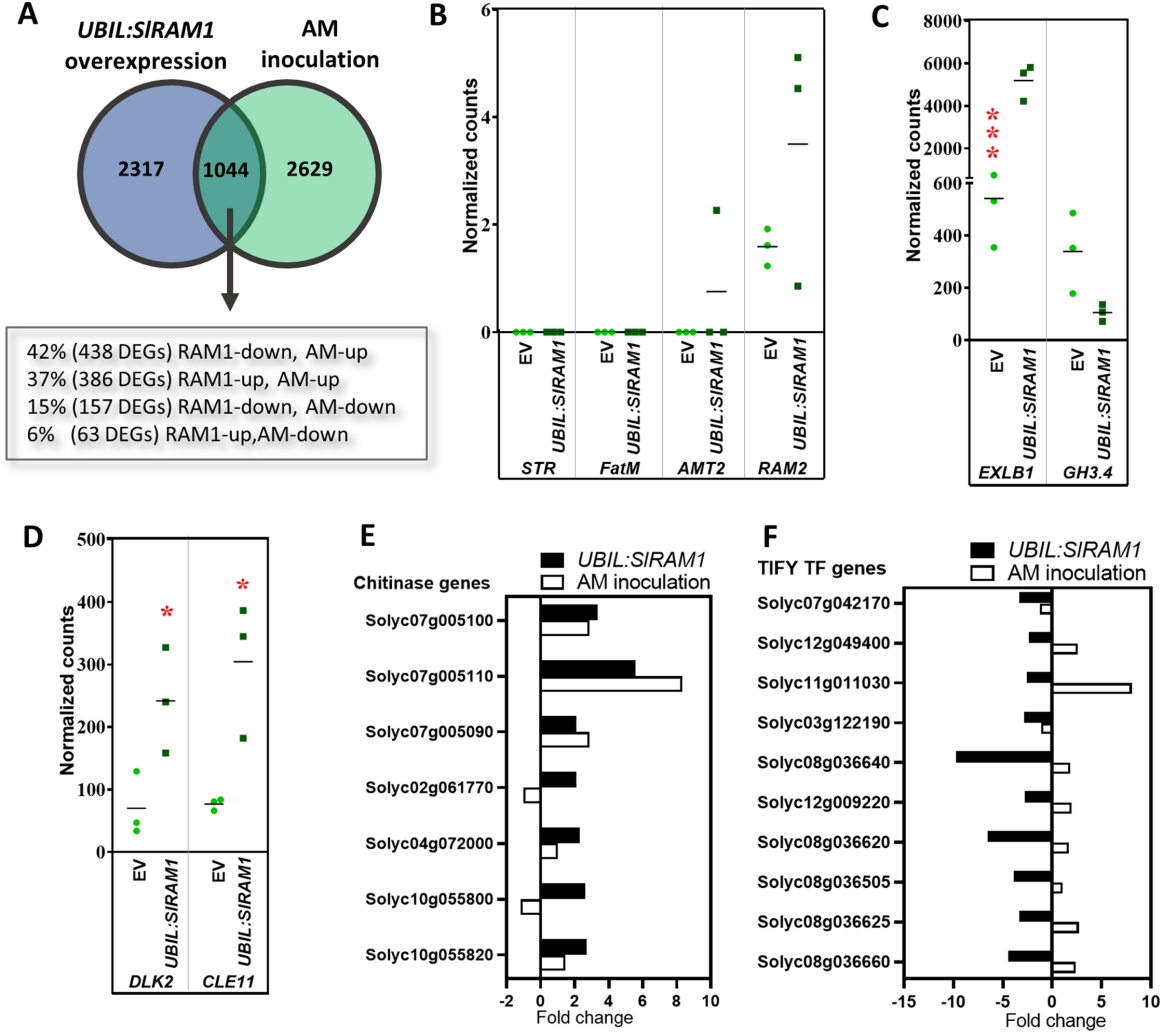
*SlRAM1* overexpression regulates arbuscular mycorrhizal (AM)-related genes in non-mycorrhizal roots. RNA-seq analysis was performed on the root tissue from 54-day-old non-mycorrhizal *UBIL:SlRAM1* composite tomato plants and control plants transformed with the empty vector (EV). (A) Venn diagram depicting the number of differentially expressed genes (DEGs) (*P*<0.05 and fold change >2 or < –2) upon either *UBIL:SlRAM1* (this work) or mycorrhization (PRJNA509606). For the 1044 genes commonly regulated by *UBIL:SlRAM1* and mycorrhization, the percentage and number of DEGs up- or down-regulated by *UBIL:SlRAM1* (RAM1-up or RAM1-down, respectively) or by AM inoculation (AM-up or AM-down, respectively) is indicated. (B–D) Expression of AM-related genes in response to *UBIL:SlRAM1*, expressed as normalized counts by DEseq and indicating significant differences (Student´s *t*-test) between the mutant and the control with asterisks (*P<0.05; ***P<0.001): (B) marker genes of arbuscule functionality (*STR*, *FatM*, *AMT2*, and *RAM2*); (C) *EXLB1* and *GH3.1*; and (D) negative AM regulators (*DLK2* and *CLE11*). (E) Overall repression of chitinases, and (F) general repression of many TIFY transcription factor genes by *UBIL:SlRAM1*.

To examine the transcriptional regulatory role of SlRAM1, we decided to analyse global transcriptional changes in tomato (non-inoculated) roots in response to *SlRAM1* overexpression. We used 54-day-old tomato composite plants transformed with the *UBIL:SlRAM1* vector, showing an 86 (±20)-fold *SlRAM1* gene expression with respect to the control plants transformed with the empty vector (EV), measured by RT–qPCR. The resulting transcriptomic data of genes regulated by *SlRAM1* overexpression were compared with a previous RNA-seq analysis of genes regulated by mycorrhization (PRJNA509606) (Ho-Plágaro *et al.*, 2019, 2021) (Supplementary Fig. S2; Supplementary Tables S3, S4). A total of 3361 genes were differentially expressed in *UBIL:SlRAM1* roots with respect to the control roots transformed with the EV, and 31% of them (1044 genes) were also significantly regulated by mycorrhization (Fig. 3A; Supplementary Table S4D), supporting the importance of SlRAM1 in the regulation of AM-related genes. In particular, when examining the 1044-set of genes commonly regulated by *UBIL:SlRAM1* and mycorrhization, we observed that the majority of these genes were AM induced (79% versus 21% down-regulated genes). However, the primary impact of *SlRAM1* overexpression on these AM-regulated genes was a down-regulation (57% versus 43% up-regulated genes) (Fig. 3A; Supplementary Table S4D).

To enhance the reliability of our results, we conducted a comparative analysis of the 3361 genes that were differentially expressed in the *UBIL:SlRAM1* roots with the 4707 genes differentially expressed upon mycorrhization from a recently reported tomato AM-associated transcriptome study (Zeng *et al.*, 2023). The analysis revealed that from all the genes differentially expressed in *UBIL:SlRAM1* roots compared with control roots transformed with the EV, 979 of these genes were also significantly regulated by mycorrhization in the study of Zeng *et al.* (2023) (Supplementary Table S4E). Upon examining this 979-set list, we observed a distribution pattern similar to that of the group of 1004 genes commonly regulated by *UBIL:SlRAM1* and mycorrhization in our study (Supplementary Fig. S3). Of these 979 genes, 65% were AM-up-regulated 0genes versus 35% AM-down-regulated, and the effect of *SlRAM1* overexpression on these AM-regulated genes was a down-regulation (65% versus 35% up-regulated), as occurred with the 1044-set list of genes described in our study. The results from both datasets showed that a set of 383 genes are co-regulated by AM and *SlRAM1* (Supplementary Fig. S3), and that the primary effect of *SlRAM1* overexpression on AM-related genes was the down-regulation of AM-induced genes.

When comparing the list of genes co-upregulated between *S. lycopersicum* L. and *L. japonicus* L. during arbuscular mycorrhizal development published by Sugimura and Saito (2017) with the list of the 1044-gene set commonly regulated in this study (Supplementary Table S4D), only a few genes were found to be commonly regulated. However, this small group of genes conserved for arbuscular mycorrhizal symbiosis encodes proteins with putative regulatory roles during mycorrhization (*FAD-binding Berberine family protein*; *Kin2*; *DLK2*; *Dirigent protein*; *CCD8*; *Leucine-rich repeat protein kinase*; *Receptor protein kinase*; *Fatty acid hydroxylase*).

**Fig. 4.**
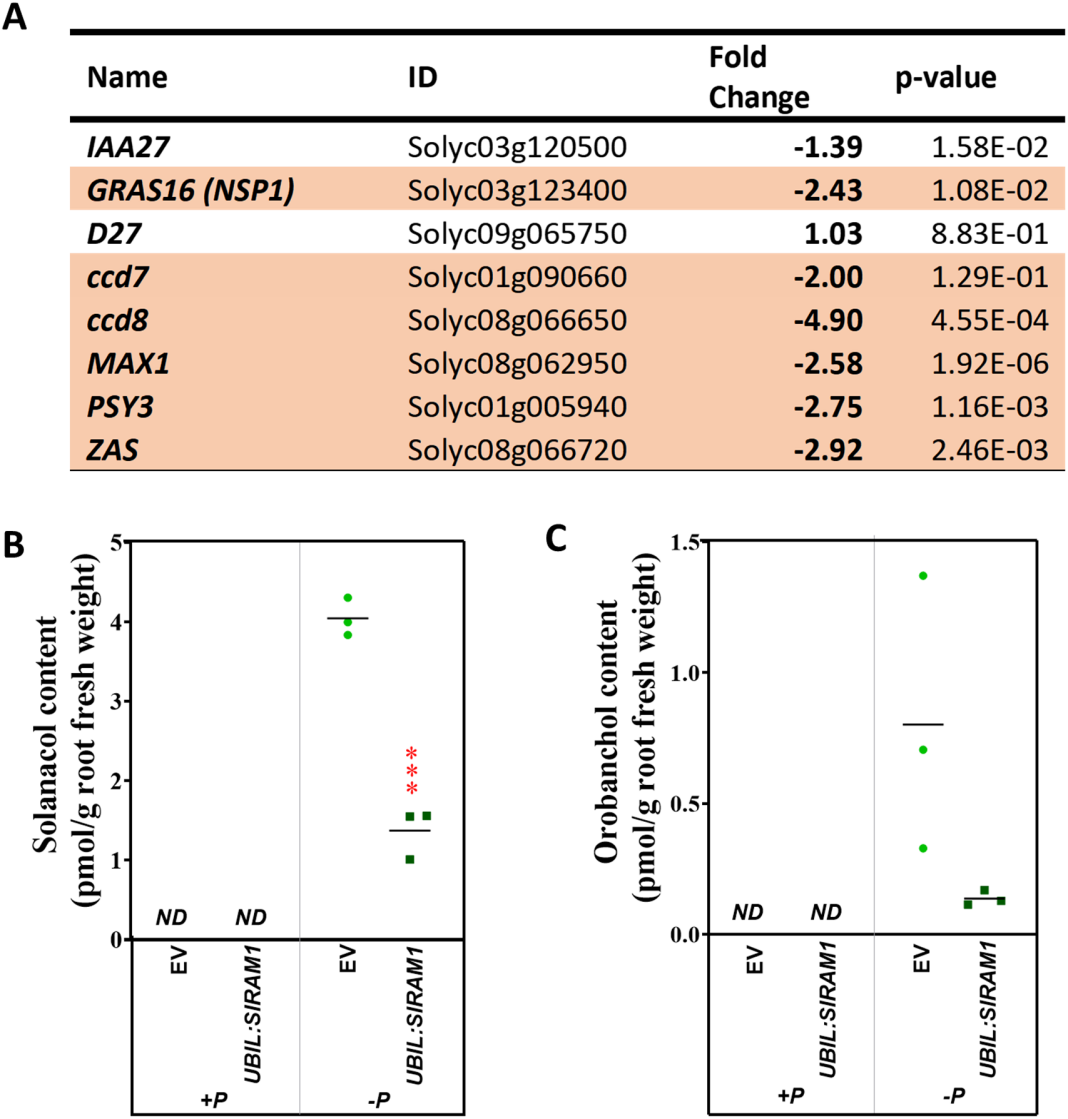
Overexpression of *SlRAM1* in roots leads to a reduction in strigolactone levels. (A) RNA-seq fold change expression and *P*-values of genes related to strigolactone biosynthesis in *UBIL:SlRAM1* with respect to control roots transformed with the empty vector (EV). (B, C) Solanacol and orobanchol contents, respectively, in non-colonized control (EV) and *UBIL:SlRAM1* roots under standard (+P) and P-starvation conditions (–P) (*n*=3). ND, not detected. Significant differences (Student’s *t*-test) with respect to plants transformed with the empty vector (EV) are indicated with asterisks (***P<0.001).

As expected, the putative homologue of RAM2 in tomato showed an induction trend in *UBIL:SlRAM1* roots, that was significant when we confirmed the result by RT–qPCR analysis (Supplementary Fig. S4). This observation is in agreement with the induction of *RAM2* when *RAM1* is constitutively expressed in *Medicago* and *Lotus* (Pimprikar *et al*., 2016; Luginbuehl *et al*., 2017), and with the direct binding of the RAM1 TF to the *RAM2* promoter previously reported in *Medicago* (Gobbato *et al.*, 2012). However, to our surprise, *UBIL:SlRAM1* roots did not show an altered expression of other typical AM marker genes of arbuscule functionality reported to be up-regulated by *RAM1* ectopic expression in non-mycorrhizal roots from other plant species, such as *STR*, *FatM*, or *AMT2* (Fig. 3B) (Park *et al.*, 2015; Pimprikar *et al*., 2016; Müller *et al.*, 2020). Nevertheless, a full examination of AM-related genes in our RNA-seq data allowed us to identify several genes differentially regulated in *UBIL:SlRAM1* roots with contrasting described functions in the AM symbiosis. For example, supporting the positive role of *SlRAM1* in mycorrhization, we observed that *SlRAM1* overexpression in tomato roots significantly induced *EXLB1*, an expansin gene required for correct root colonization, arbuscule expansion, and AM formation (Dermatsev *et al.*, 2010), and seemed to repress the *GH3.4* gene, which is induced during mycorrhization and negatively regulates mycorrhization via maintaining cellular auxin homeostasis (Chen *et al.*, 2022) (Fig. 3C). On the other hand, some of the identified transcriptional changes occurring in the *UBIL:SlRAM1* roots pointed towards a negative regulation of mycorrhization. In this sense, *SlRAM1* overexpression induced transcriptional levels of *SlDLK2* and *SlCLE11* (Fig. 3D), already characterized in tomato as AM-negative regulators (Ho-Plágaro *et al.*, 2021; Wulf *et al.*, 2023, Preprint), and, as shown later in Fig. 4A, *UBIL:SlRAM1* triggered the repression of genes related to biosynthesis of SL, a plant hormone essential for pre-symbiotic signalling and efficient hyphal entry into the roots (Akiyama *et al.*, 2005; Kobae *et al.*, 2018). Moreover, Gene Ontology (GO) analyses revealed that dataset genes induced and repressed by *UBIL:SlRAM1* are over-represented in GO terms that are also enriched during mycorrhization (Supplementary Table S5). GO terms commonly over-represented among the *UBIL:SlRAM1-*induced and AM-induced gene sets are the ‘amino sugar catabolic process’, ‘chitinase activity’, ‘hormone binding’, ‘inorganic anion transmembrane transporter activity’, and ‘cell wall’ and ‘extracellular region’ localizations; as well as ‘peroxidase’, ‘protease’, and ‘phosphatase’ protein classes. Of particular importance is the ‘chitinase activity’ GO term, which showed a 10.5-fold enrichment in the *UBIL:SlRAM1-*induced gene set, unravelling an overall induction of AM-induced chitinases (Fig. 3E). On the other hand, we also found several GO terms enriched in the *UBIL:SlRAM1*-repressed gene set that were also over-represented in the AM-induced genes, such as ‘response to wounding’, ‘regulation of defense response’, ‘regulation of stress response’, ‘lipid metabolic process’, ‘cellular response to lipid’, ‘organic hydroxy compound metabolic process’, ‘cellular response to organic substance’, and ‘plant-type vacuole membrane’ localization, as well as ‘hydrolase’ and ‘lipase’ protein classes (Supplementary Table S5). The ‘response to wounding’ and ‘response to defense response’ GO terms were the most enriched (11.6-fold enrichment) in the *UBIL:SlRAM1-*repressed genes, and were mainly associated with a general repression of many TIFY TFs (Fig. 3F), which have been related to stress responses and are the second most induced family of TFs during mycorrhization in tomato roots (Chini *et al.*, 2017; Ho-Plágaro *et al.*, 2019).

### Strigolactone biosynthesis repression partially contributed to the impaired mycorrhizal colonization in *UBIL:SlRAM1* roots

Our RNA-seq data revealed that *SlRAM1* overexpression leads to a decrease expression of several genes related to SL biosynthesis (Fig. 4A). In particular, *CCD7*, *CCD8*, and *MAX1*, encoding enzymes from the SL biosynthetic pathway, were repressed. Moreover, we observed a repression of tomato homologues of genes with a reported function in the SL biosynthesis in other species, such as a phytoene synthase 3 (*PSY3*), catalysing the first step in carotenoid biosynthesis and determining SL levels (Stauder *et al.*, 2018), a putative zaxinone synthase (*ZAS*), involved in the control of SL levels (Wang *et al.*, 2019), and the putative tomato *NSP1* TF gene, required for SL biosynthesis (Liu *et al.*, 2011; Takeda *et al.*, 2013).

To confirm if SL contents were reduced upon *SlRAM1* overexpression, as suggested by the RNA-seq data, we performed an experiment where *UBIL:SlRAM1* and control plants were subjected to P-starvation conditions to promote SL production. Effective *SlRAM1* overexpression and the induction of the P-starvation marker gene *SlTPSI1* (Liu *et al.*, 1997) under P-deficient conditions were validated by RT–qPCR (Supplementary Fig. S5). As expected, SLs were not detected in the control treatment, while an increase in two types of SLs (solanacol and orobanchol) was observed under P-starvation conditions (Fig. 4B and C, respectively). Moreover, SL levels in the *UBIL:SlRAM1* roots under P-deficient conditions were lower than in the control roots, showing a significant decrease in the case of solanacol (Fig. 4B), which is one of the most abundant SLs in tomato (Kohlen *et al.*, 2013). These results confirm that *SlRAM1* overexpression causes a reduction of SL contents in roots.

**Fig. 5.**
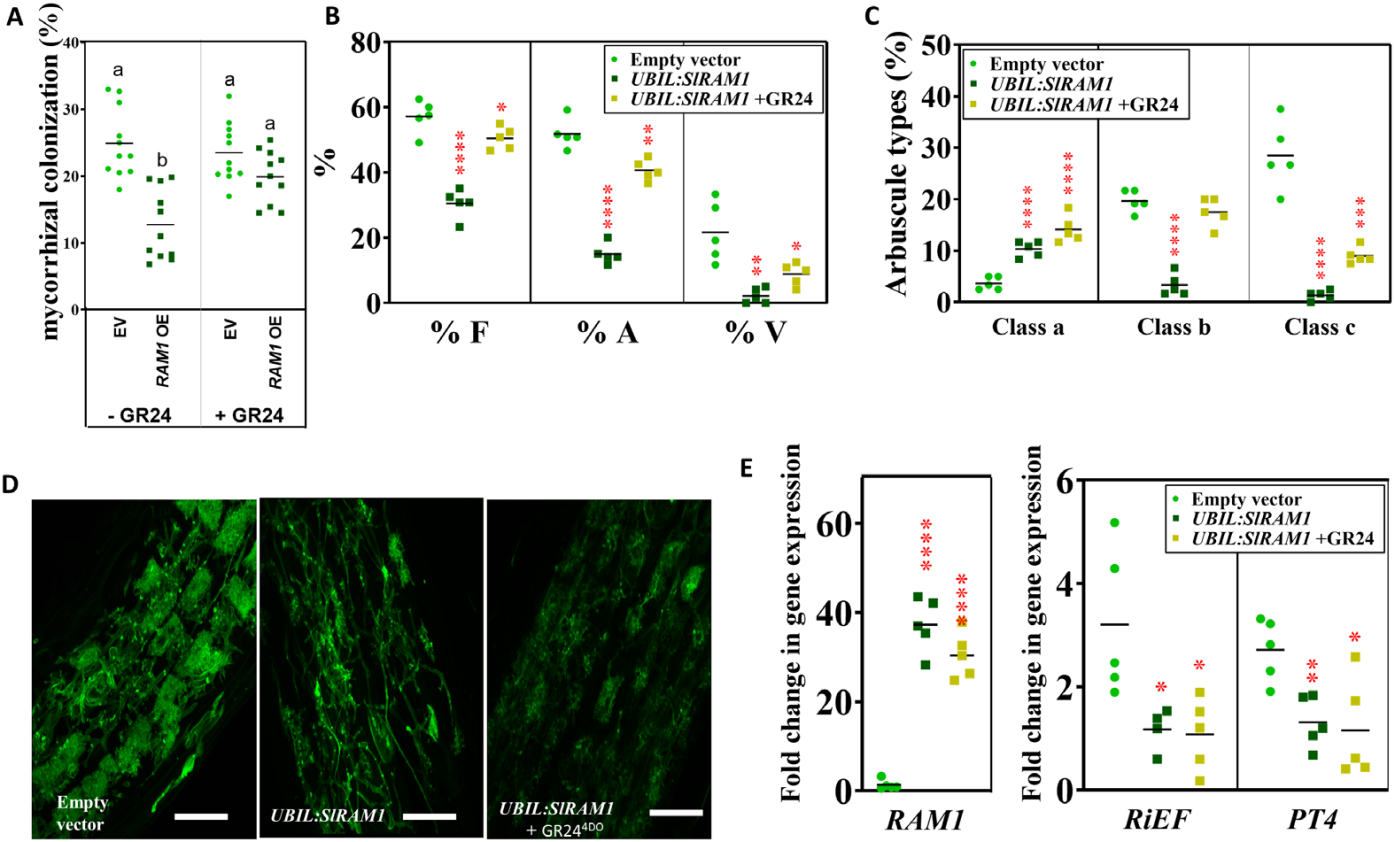
Strigolactone biosynthesis partially contributed to the impaired mycorrhizal colonization in *UBIL:SlRAM1* roots. (A) Colonization levels observed in control (EV) and *UBIL:SlRAM1* 60-day-old hairy root plants, not treated (–GR24) or supplemented by the external application of 25 ml of the strigolactone analogue GR24^4DO^ at 0.05 µM twice a week (+GR24) (*n*=11). Five samples from control [empty vector (EV)], *UBIL:SlRAM1*, and *UBIL:SlRAM1* hairy roots supplemented with GR24^4DO^ were randomly selected for further analyses. (B) Mycorrhizal parameters: frequencies of mycorrhization (%F), arbuscules (%A), and vesicles (%V) in hairy root genotypes. (C) Categorization and prevalence of small-, middle-, or full-sized arbuscules (classes a, b, and c, respectively) among mycorrhizal hairy root genotypes. (D) Representative images of mycorrhizal colonization in roots subjected to WGA–Alexa Fluor 488 staining and visualized by confocal laser scanning microscopy (scale bars=75 μm). Fully developed arbuscules were easily observed in hairy roots transformed with the EV, while a higher proportion of underdeveloped arbuscules were found in *UBIL:SlRAM1* roots. Partial recovery of middle- or full-sized arbuscules was observed in *UBIL:SlRAM1* hairy roots supplemented with GR24^4DO^. (E) Expression of *SlRAM1*, *SlPT4*, and *RiEF* genes measured by RT–qPCR. Gene expression data were represented with respect to EV (in the case of *SlRAM1*) or mycorrhizal *UBIL:SlRAM1* roots (in the case of *SlPT4* and *RiEF*), for which the expression level was designated as 1. Significant differences (Student’s *t*-test) with respect to plants transformed with the EV are indicated with asterisks (*P<0.05; **P<0.01; ***P<0.001; ****P<0.0001). *PT4*=*Phosphate transporter 4*; *RAM1*=*Required for Arbuscular Mycorrhization 1*; *RiEF*=*R. irregularis Elongation factor*.

Finally, we performed a mycorrhizal experiment with *UBIL:SlRAM1* composite tomato plants supplemented or not with the SL analogue stereoisomer GR24^4DO^. It was observed that the negative effect of *SlRAM1* overexpression on colonization levels did not occur in plants submitted to the GR24^4DO^ treatment, supporting the hypothesis that one of the mechanisms for the negative regulation of the AM symbiosis by the SlRAM1 TF is mediated by a repression of SL biosynthesis, which leads to a reduced AM colonization (Fig. 5A). In this experiment, a dose of GR24^4DO^ was utilized, carefully calibrated according to bibliographic data (Müller *et al.*, 2019; Lidoy-Logroño, 2023), to avoid influencing the mycorrhization of EV plants (possessing higher basal levels of SLs), while still being adequate to offset the SL deficiency in *UBIL:SlRAM1* composite plants.

The pronounced deregulation of symbiosis in the *UBIL:SlRAM1* roots (Fig. 2) is unlikely to be controlled solely by the observed decrease in SL levels in roots. Therefore, we conducted a thorough investigation of the symbiotic phenotype in control plants, in plants overexpressing *SlRAM1*, and in composite *SlRAM1* OE plants treated with GR24^4DO^. The analysis of mycorrhization, arbuscular, and vesicle frequencies clearly indicated a substantial recovery effect (~90%) in the frequency of colonization with the addition of GR24^4DO^. The arbuscular frequency recovered to 75%, while that of vesicles only reached 40% with the exogenous addition of synthetic SL (Fig. 5B). However, upon detailed analysis of the recovery of arbuscular colonization, specifically through arbuscular categorization, a distinct pattern was observed for each of the hairy root genotypes studied. In control plants, the arbuscular pattern was characterized by a higher percentage of medium and well-developed types, compared with a low proportion of small, poorly developed arbuscules (Fig. 5C, D). As observed in previous experiments (Fig. 2C, D), *UBIL:SlRAM1* roots exhibited a significant decrease in the percentage of medium- and large-sized arbuscules, alongside a higher proportion of underdeveloped ones (Fig. 5C, D). The application of GR24^4DO^ to *UBIL:SlRAM1* plants contributed to a redistribution of the arbuscular pattern compared with non-treated *UBIL:SlRAM1* plants, although without recovering the pattern observed in control plants, showing an equal proportion of immature and medium arbuscules and a low proportion of well-developed arbuscules, reaching only 25% of the proportion found in control plants (Fig. 5C, D). Successful up-regulation of *SlRAM1* gene expression in the *UBIL:SlRAM1* hairy roots was confirmed and, when analysing the expression pattern of *SlPt4*, a marker gene for arbuscular activity, and *RiEF* among the different hairy root genotypes (Fig. 5E), no recovery of *Pt4* and *RiEF* transcription rates in *UBIL:SlRAM1* roots treated with GR24^4DO^ was observed.

## Discussion

Although emerging evidence suggests that many GRAS factors operate as TFs and TRs in complexes with other GRAS proteins regulating AM symbiosis at different levels, their functional significance for AM symbiosis is only partially known. Despite this lack of knowledge, some remarkable findings suggest the existence of interconnected transcriptional modules regulated by multiple GRAS transcription factors whose participation is essential for the transcriptional reprogramming in the host plant underlying the morphogenetic adaptive changes in roots leading to the formation, function, and degeneration of the arbuscules (reviewed by Ho Plágaro and García-Garrido, 2022).

RAM1 stands out among all GRAS proteins involved in AM symbiosis regulation. RAM1 is a major regulator of the cortical cell transcriptional programme leading to arbuscule development and bidirectional nutrient exchange between host root plant cells and AM fungi (Pimprikar *et al*., 2016; Luginbuehl *et al*., 2017; Rich *et al*., 2017). RNA-seq studies performed in *ram1* mutants in roots from *Medicago* and petunia (Luginbuehl *et al*., 2017; Rich *et al*., 2017) show a defective up-regulation of several AM-induced genes upon mycorrhization, including *RAM2*, *PT4*, *STR*, *WRI5a*, and *FatM*. However, we cannot discard a certain degree of diversification of RAM1 functions in the AM symbiosis among plant species during evolution because, for example in legumes, there is evidence of a species-specific micro-diversification of the RAM1 and RAD1 relative contributions to arbuscule development and symbiotic gene expression (Park *et al.*, 2015; Xue *et al.*, 2015; Pimprikar *et al*., 2016; Pimprikar and Gutjahr, 2018), and in the monocot *B. distachyon*, diversification of the RAM1 function might also explain the milder arbuscule development phenotype of *ram1* mutants (Müller *et al.*, 2020). Therefore, knowledge concerning specific functions of RAM1 and other TFs in the control of AM symbiosis in a particular plant species becomes essential to design approaches directed towards an increased symbiotic efficiency mediated by manipulation of TF gene expression.

We previously identified the *GRAS27* gene in tomato as the homologue of the GRAS TF gene *RAM1* (*SlRAM1*) (Ho-Plágaro *et al.*, 2019). Similar to orthologous *RAM1* genes of *M. truncatula* (Gobbato *et al.*, 2013; Park *et al.*, 2015), *L. japonicus* (Xue *et al.*, 2015; Pimprikar *et al*., 2016), *P. hybrida* (Rich *et al*., 2015), and *B. distachyon* (Müller *et al.*, 2020), *SlRAM1* expression is induced in mycorrhizal roots and expressed in arbuscule-containing cells (Ho-Plágaro *et al.*, 2019). Here, to further determine the symbiotic role of SlRAM1, a functional analysis with *SlRAM1* RNAi hairy root composite tomato plants was performed. Concerning the control roots transformed with the EV, *SlRAM1* RNAi hairy roots showed a compromised AM colonization, and stunted arbuscules appeared in the cortex (Fig. 1). This indicates that SlRAM1 is required for arbuscule development, which was consistent with the RAM1 function described in other plant species (Park *et al.*, 2015; Xue *et al.*, 2015).

Given the *SlRAM1* AM-inducible expression and its pivotal regulatory role in tomato roots, we hypothesized that overexpression of *SlRAM1* might increase the expression of several AM-induced genes and the occurrence of arbuscules in colonized roots. To test this, two parallel scientific approaches were carried out. First, we assayed whether *SlRAM1* overexpression modifies AM colonization patterns; secondly, through an RNA-seq study, we evaluated the capability of SlRAM1 to regulate the expression of several AM-related genes in the absence of symbiosis when *SlRAM1* was ectopically overexpressed in tomato roots.

Although composite plants with hairy roots may exhibit some level of variation due to positional effects at the vector integration site, and overexpression could potentially trigger gain-of-function effects or alter the transcriptional network unrelated to the gene’s normal function, numerous studies utilizing ectopic gene expression have been employed to functionally characterize the role of GRAS transcription factors during mycorrhization (Park *et al.*, 2015; Heck *et al.*, 2016; Müller *et al.*, 2020) or to conduct RNA-seq experiments aiming to identify differentially expressed genes regulated by the ectopic expression of a target gene in hairy roots (Sun *et al.*, 2016; Zhang *et al.*, 2017). These studies have included analyses in the roots of tomato and *M. truncatula* mycorrhizal composite plants (Müller *et al.*, 2019; Ho-Plágaro *et al*., 2021).

Unexpectedly, when overexpressing *SlRAM1* under the control of the maize ubiquitin promoter, *UBIL:SlRAM1* roots showed a reduced mycorrhizal colonization (Fig. 2), and this negative effect seemed to be accentuated at later stages of mycorrhization and was accompanied by a decreased proportion in the presence of developed and mature arbuscules. By contrast, a higher relative abundance of root intersects with only hyphae or initiating arbuscules was found in the *UBIL:SlRAM1* roots than in the control roots, suggesting a delay in mycorrhizal dynamics in the *UBIL:SlRAM1* roots. Moreover, a general repression of several AM marker genes involved from early (SL biosynthesis and CSSP genes) to more advanced stages of the interaction (arbuscule functionality genes) was observed in the *UBIL:SlRAM1* roots. However, we should note that this effect might be due to the general decreased mycorrhizal levels of the *UBIL:SlRAM1* roots, and not to a direct repression of these genes by the SlRAM1 TF. The apparent contradictory results obtained in the *SlRAM1* RNAi and *UBIL:SlRAM1* plants suggest that SlRAM1 could have positive and negative roles, respectively, on mycorrhization.

To identify targets of SlRAM1 on AM-related genes, interference effects of mycorrhization were avoided by performing an RNA-seq experiment where *SlRAM1* was ectopically expressed in non-mycorrhizal roots (Fig. 3).

In this study, one-third of the genes regulated by *SlRAM1* overexpression were also regulated by AM, supporting the key role of RAM1 as a master TF in AM symbiosis. However, surprisingly, among the AM and *UBIL:SlRAM1* commonly regulated genes, we did not find several typical AM marker genes reported to be up-regulated by *RAM1* overexpression in other plant species, such as *FatM*, *AMT2*, and *STR* (Park *et al.*, 2015; Pimprikar *et al*., 2016; Müller *et al.*, 2020). Comparative analysis using previous AM-associated transcriptomic data from *Lotus* and tomato (Sugimura and Saito, 2017) showed that only a few genes were co-regulated in both species and by *SlRAM1* ectopic overexpression in tomato roots. This low ratio of co-regulation could be due to the fact that only one-quarter to one-third of the AM-inducible genes in each plant species were co-up-regulated in both species (Sugimura and Saito, 2017), and also to differences in the inoculation process, the days after inoculation, and/or the plant species and the colonization and growth status. However, this small group of genes are conserved for AM symbiosis and encode proteins with putative regulatory roles during mycorrhization. On the other hand, the comparative analysis of our data with data from a recently reported tomato AM-associated transcriptome study (Zeng *et al.*, 2023) demonstrated that the sets of AM-regulated genes differentially regulated by *SlRAM1* ectopic overexpression showed a distribution pattern characterized by the down-regulation of AM-induced genes by *SlRAM1* overexpression, suggesting a putative negative regulatory role for RAM1.

AM-related genes were found to be differentially expressed upon *SlRAM1* overexpression in tomato and, in agreement with the mycorrhizal phenotypes observed in the *SlRAM1* RNAi and OE roots, the observed transcriptomic changes supported a dual role for SlRAM1 on mycorrhization. A positive role for SlRAM1 on mycorrhization was indicated by the induction of *RAM2* upon *SlRAM1* overexpression, also reported in *Medicago* and *Lotus* (Pimprikar *et al*., 2016; Luginbuehl and Oldroyd, 2017), and the induction of other tomato genes required for a correct colonization such as the expansin *EXLB1* (Dermatsev *et al.*, 2010). Moreover, although *UBIL:SlRAM1* roots did not show an induction of arbuscule transporter genes such as *AMT2* and *PT4* induced by *RAM1* in *Lotu*s (Pimprikar *et al*., 2016), GO analyses suggest that, similarly, in tomato SlRAM1 might promote mycorrhization through up-regulation of other genes related to ‘inorganic anion transmembrane transporter activity’, as this GO term is enriched by *SlRAM1* overexpression (Supplementary Table S5). On the other hand, the up-regulation of negative regulators of mycorrhization (*SlDLK2* and *CLE11*), the repression of SL biosynthetic genes, the induction of many plant chitinases that are commonly considered to have an inhibitory effect on fungal hyphal growth (Vierheilig *et al.*, 2001), and the overall down-regulation of AM-induced genes observed by *SlRAM1* overexpression support the negative role of SlRAM1 in mycorrhization (Fig. 3).

Although the negative effect of *SlRAM1* overexpression on mycorrhization through the regulation of these targets should be experimentally confirmed, we decided to focus our attention on the down-regulation of SL biosynthesis-related genes in the *UBIL:SlRAM1* roots as one of the possible mechanisms. SLs are apocarotenoid-derived phytohormones with diverse functions in plants (Gomez-Roldan *et al.*, 2008; Umehara *et al.*, 2010), that also serve as signals that are secreted into the rhizosphere in response to Pi deficiency conditions, indicating to AM fungi the presence of a receptive host to be colonized (Yoneyama *et al.*, 2007). In particular, secreted SLs promote fungal spore germination, as well as hyphal growth and branching, and then increase the possibilities of physical contact of the AM fungus with the roots (Akiyama *et al.*, 2005; Besserer *et al.*, 2006). Curiously, Müller *et al.* (2020) previously observed that RAM1 ectopically expressed in non-colonized roots of the monocot *B. distachyon* also triggered a down-regulation of genes involved in the SL biosynthesis pathway (*BdD27*, *BdCCD7*, and *BdCCD*) relative to control roots, and they explained this effect as possible native functions of BdRAM1 in hormone signalling or a misregulation of the SL biosynthetic pathway by the disruption of GRAS factor complexes by *RAM1* overexpression. However, the authors of the study did not contemplate the possibility of an effect of RAM1 as a negative regulator of mycorrhization through a repression of SL biosynthesis, consequently inhibiting pre-symbiotic signalling. We confirmed that SL contents diminished in *UBIL:SlRAM1* roots under Pi limitations, and we tested this hypothesis in a mycorrhizal experiment (Fig. 5). Effectively, treatment of plants with the SL analogue stereoisomer GR24^4DO^ restored the decreased percentage of mycorrhizal colonization in roots of *UBIL:SlRAM1* composite plants, supporting a role for SlRAM1 in the control of mycorrhization through a repression of SL biosynthesis and an inhibition of pre-symbiotic signalling. Interestingly, López-Ráez *et al.* (2015) reported that SL biosynthesis is reduced when the AM fungus is well stablished in the root, and pointed to the presence of autoregulatory mechanisms to avoid overcolonization once the plant has reached a better nutritional status. In this respect, our data strongly suggest that the RAM1 TF might be a key component in this autoregulatory process.

However, an in-depth study of the mycorrhizal phenotype of these overexpressing plants, whether subjected to exogenous supply of GR24^4DO^ or not, showed that while the mycorrhizal frequency recovered almost entirely, the same did not occur with arbuscular colonization. The data indicated that the addition of the synthetic SL did not reverse the negative effect of *SlRAM1* overexpression on arbuscular development, suggesting that this negative effect is not only exerted through SLs, and that other mechanism(s) may be involved.

**Fig. 6.**
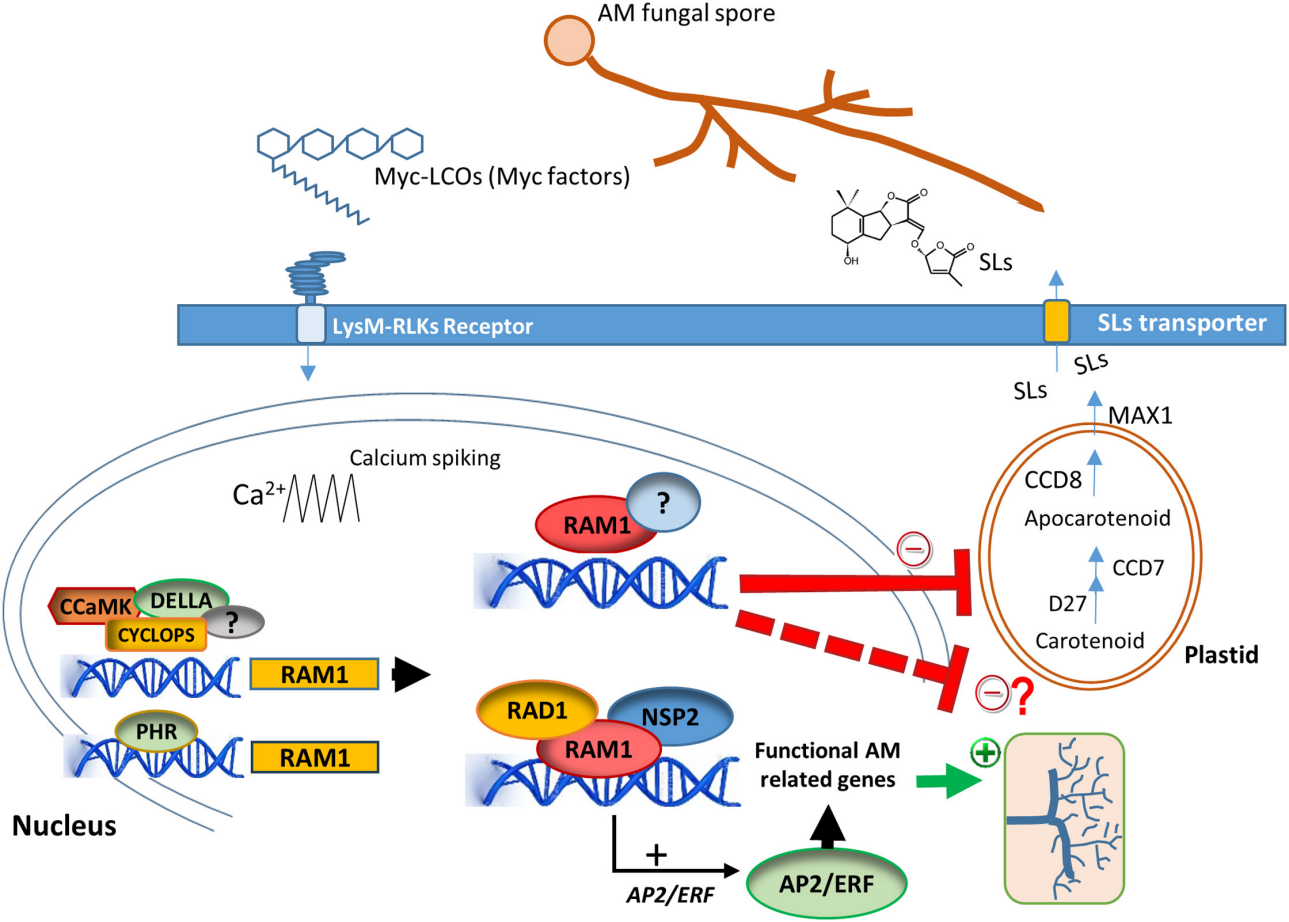
Participation of RAM1 in the regulation of arbuscular mycorrhizal (AM) formation. The release of strigolactones (SLs) in the rhizosphere induces spore germination and hyphal branching, along with the secretion of mycorrhizal factors (Myc factors) from AM fungi. Perception of Myc factors by Lys-RLK receptors triggers the symbiotic pathway, including the induction of calcium oscillations in the nucleus, leading to the activation of CCaMK and CYCLOPS. In a complex with DELLA proteins, CYCLOPS activates the expression of *RAM1*. Alternatively, *RAM1* expression can be also induced by Pi-starvation conditions through PHR TFs. RAM1, capable of interacting with several other GRAS-domain proteins such as RAD1 and NSP2, activates the expression of genes involved in arbuscule development and functionality. These genes include those for lipid biosynthesis and the control of nutrient transfer in the periarbuscular membrane, probably through the activation of other TFs, including AP2/ERF TFs. Furthermore, in tomato, RAM1 directly down-regulates gene expression of apocarotenoid and SL biosynthesis genes, including *ccd8* and *max1*, thereby reducing SL release and minimizing rhizosphere signalling. This suggests that the RAM1 transcription factor might be a key component in the autoregulatory process of mycorrhization. Additionally, a negative effect of *SlRAM1* overexpression on arbuscular development, independent of the decrease in strigolactone release, can be envisaged (proposed unknown mechanism represented by a dashed line). Abbreviations: AP2/ERF, Apetala/ethylene Responsive Factor; CCD, carotenoid cleavage dioxygenase; LysM-RLKs, LysM-containing receptor-like kinases; Myc-LCOs, mycorrhizal lipochitinoligosaccharides; PHRs, phosphate starvation response; RAD1, Required for Arbuscule Development 1; RAM1, Required for Arbuscular Mycorrhization 1.

Overall, our results indicate a functional diversification of the regulatory cascade responsible for the transcriptional reprogramming of roots during AM symbiosis in plant species. We discovered that, in tomato, SlRAM1 may not only play a positive role in mycorrhization but, could also fulfil novel negative regulatory roles in mycorrhization, probably associated with, among other mechanisms, the repression of SL biosynthesis (Fig. 6). Future experiments will aim to unveil additional mechanisms responsible for the negative regulation of mycorrhization by SlRAM1, as well as to elucidate potential interacting partners and RAM1 complexes that determine the positive and negative regulatory roles of this TF in mycorrhization.

## Supplementary data

The following supplementary data are available at *JXB* online.

Fig. S1. Mycorrhizal colonization in *UBIL:SlRAM1* roots inoculated with *Funneliformis mosseae*.

Fig. S2. Validation of RNA-seq data analysis by RT–qPCR.

Fig. S3. Differentially regulated genes by *UBIL:SlRAM1*.

Fig. S4. *SlRAM2* gene expression in *UBIL:SlRAM1* hairy roots of composite plants.

Fig. S5. Expression analysis of *SlRAM1* and *SlTPSI1* upon *UBIL:SlRAM1* and P-starvation conditions.

Table S1. Primers used in this study for RT–qPCR experiments.

Table S2. MRM conditions for endogenous strigolactones and GR24.

Table S3. Number of mapped reads, high quality reads, and splice reads for libraries from each sample in the RNA-seq analysis.

Table S4. List of DEGs generated by RNA-seq found to be differentially expressed upon *UBUL:SlRAM1* expression in non-inoculated roots and by AM inoculation.

Table S5. Gene ontology and protein class analyses on *UBIL:SlRAM1*-induced and repressed genes.

erae210_suppl_Supplementary_Figures_S1-S5_Tables_S1-S3_S5

erae210_suppl_Supplementary_Table_S4

## Data Availability

All data supporting the findings of this study are available within the paper and within its supplementary data published online. The raw sequence reads generated in this and previous studies were deposited in the NCBI Sequence Read Archive (SRA) database with accession nos PRJNA523214 and PRJNA509606, respectively.
