# Supplementary material for: A dual regulatory role for the arbuscular mycorrhizal master regulator RAM1 in tomato
Source: J Exp Bot. 2024 May 10;75(16):5021–36. doi: 10.1093/jxb/erae210 (PMC11349867; doi:10.1093/jxb/erae210)
Supplement: erae210_suppl_Supplementary_Figures_S1-S5_Tables_S1-S3_S5 [file erae210_suppl_supplementary_figures_s1-s5_tables_s1-s3_s5.pdf]

A dual regulatory role of the arbuscular mycorrhizal master regulator RAM1 in tomato.

Tania Ho-Plágaro; María Isabel Tamayo-Navarrete; Sanja Cavar; Petr Tarkowski; José Manuel García-Garrido

The following Supplementary data is available for this article:

Fig. S1. Mycorrhizal colonization in *UBIL:SlRAM1* roots inoculated with *Funneliformis mosseae*.
Fig. S2. Validation of RNAseq data analysis by RT- qPCR.
Fig. S3. Differentially regulated genes by *UBIL:SlRAM1*.
Fig. S4. *SlRAM2* gene expression in *UBIL:SlRAM1* hairy roots of composite plants.
Fig. S5. Expression analysis of *SlRAM1* and *SlTPSI1* upon *UBIL:SlRAM1* and P-starvation conditions.
Table S1. Primers used in this study for quantitative reverse transcription polymerase chain reaction (RT-qPCR) experiments.
Table S2. MRM conditions for endogenous strigolactones and GR24.
Table S3. Number of mapped reads, high quality reads and splices reads for libraries from each sample in the RNA-seq analysis.
Table S4. List of DEGs genes. List of DEGs generated by RNA-seq found to be differentially expressed upon *UBUL:SlRAM1* expression in non-inoculated roots and by AM inoculation (Displayed as a separate excel file).
Table S5. Gene ontology and protein class analyses on *UBIL:SlRAM1* induced and repressed genes.

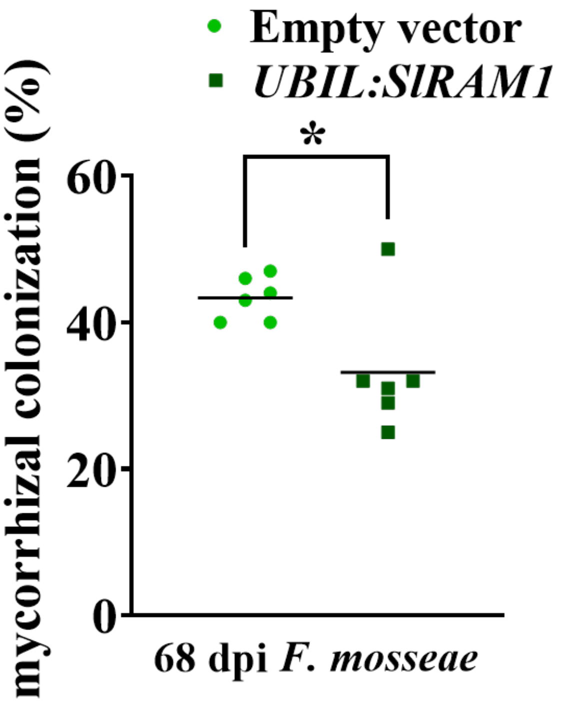

Fig. S1 Mycorrhizal colonization in *UBIL:SlRAM1* roots inoculated with *Funneliformis mosseae*. The percentage of total root length colonized was measured in the hairy root systems of *UBIL:SlRAM1* composite plants and control plants transformed with the empty vector at 68 days after inoculation with the AM fungus *F. mosseae*. Values correspond to mean ± SE (n=6). Significant difference (Student´s t-test) is indicated with an asterisk (*P<0.05).

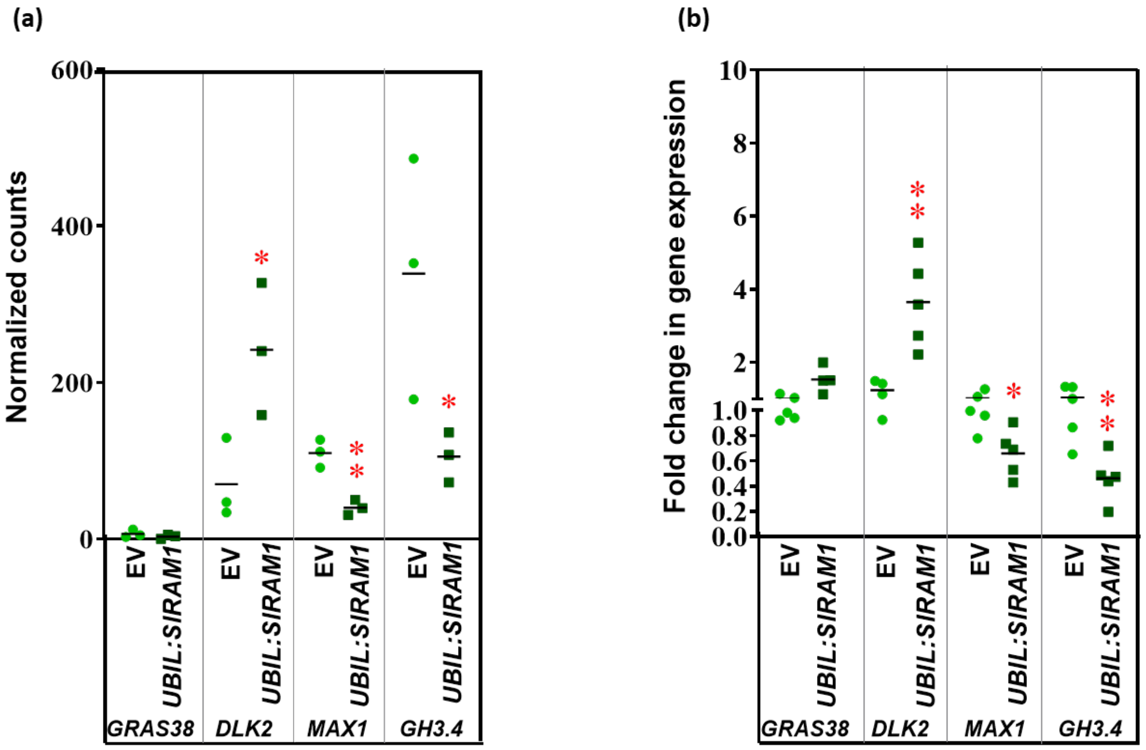

Fig. S2 Validation of RNAseq data analysis by RT- qPCR. (a) Expression level of *GRAS38*, *DLK2*, *MAX1* and *GH3.4* genes in the RNA-seq analysis (n=3). (b) Expression level of the same genes analysed by RT-qPCR using five independent biological replicates from the same experiment. Fold change in gene expression was represented with respect to the control roots transformed with the empty vector (EV). Significant differences (Student´s t test) are indicated with asterisks (**P* ≤0.05, **P ≤ 0.01).

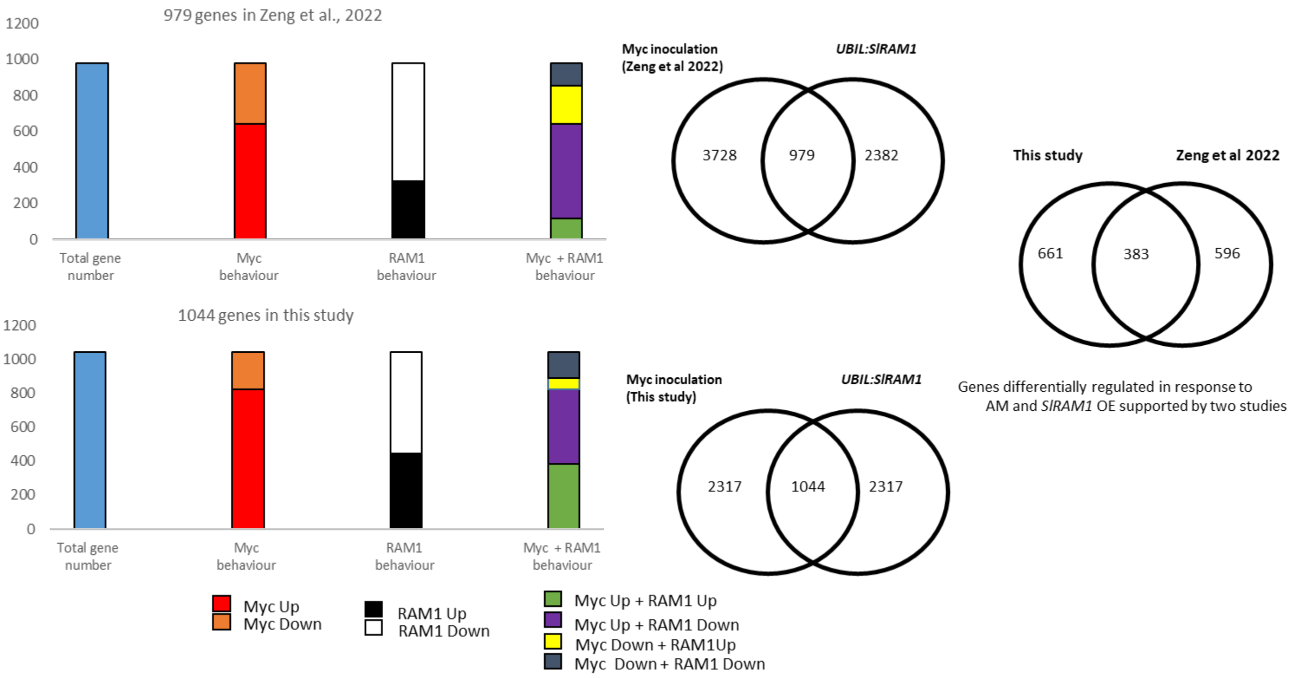

Fig. S3 Differentially regulated genes by RAM1. Diagrams showing the number of genes differentially regulated (DEGs) in response to AM and *UBIL:SlRAM1* expression among the present study and the previous published data from Zeng et al. The number of DEGs upregulated or downregulated by *UBIL:SlRAM1* expression (RAM1-up or RAM1-down, respectively) or by AM inoculation (AM-up or AM-down, respectively) is indicated.

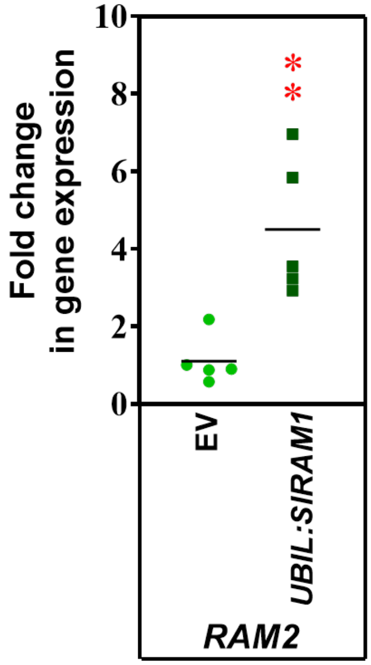

Fig. S4 *SlRAM2* gene expression in *UBIL:SlRAM1* hairy roots of composite plants. Expression level of *SlRAM2* gene was analysed by RT-qPCR with five independent biological replicates from the same experiment used for the RNA-seq. Fold change in gene expression was represented with respect to the control roots transformed with the empty vector (EV). Significant differences (Student´s t test) are indicated with asterisks (**P < 0.01).

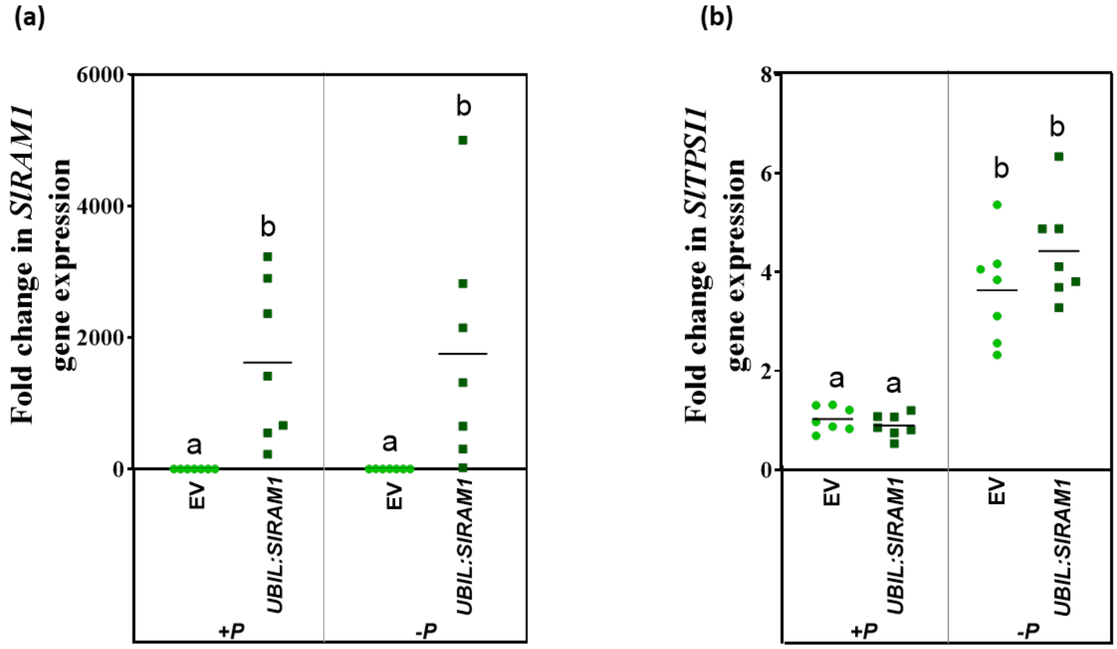

Fig. S5 Expression analysis of *SlRAM1* and *SlTPSI1* upon *UBIL:SlRAM1* expression and P-starvation conditions. The effect of *SlRAM1* overexpression (*UBIL:SlRAM1*) and phosphate deficiency (-P) on transcript levels of *SlRAM1* (a) and *SlTPSI1* (b), the tomato homolog to IPS1 (Liu *et al.*, 1997) was analysed by RT-qPCR with seven independent biological replicates. Fold change in gene expression was represented with respect to the control roots transformed with the empty vector (EV) under normal phosphate conditions (+P). Conditions with similar letters are not significantly different (P>0.05) according to Tukey's multiple comparisons test.

Table S1 Primers used in this study for quantitative reverse transcription polymerase chain reaction (RT-qPCR) experiments.

**Target sequence for cloning**	**Primer name**	**Primer sequence (5´→3´)**	**Reference**
SlRAM1 **OE**	RAM1-caccATG<br>RAM1stop-R	(5´-CACCATGGGGAGTTTAAAGAACGAC-3´)<br>(5´-TCAGCATCGCCATGCAGAGGCAG-3´)	This work
SlRAM1 **RNAi**	RAM1-caccATG<br>iRAM1-R	(5´-CACCATGGGGAGTTTAAAGAACGAC-3´)<br>(5´-GGATTTCAACATCATCATCGTCG-3´)	This work
**Target gene for qPCR [GenBank/RefSeq/SolDB accession number]**	**Primer name**	**Primer sequence (5´→3´)**	**Reference**
Rhizophagus irregularis **(Schüßler and Walker, 2010)**			
RiEF [DQ282611]	qRiEF-F<br>qRiEF-R	(5´-GCTATTTTGATCATTGCCGCC-3´)<br>(5´-TCATTAAAACGTTCTTCCGACC-3´)	Benabdellah *et al.* (2009)
Solanum lycopersicum **L**			
LeEF-1α [Solyc06g005060.2]	qLeEF1α-F<br>qLeEF1α-R	(5´-GGTGGCGAGCATGATTTTGA-3´)<br>(5´-CGAGCCAACCATGGAAAACAA-3´)	García Garrido *et al.* (2010)
SlRAM1 [Solyc02g094340.1]	qGRAS27-F<br>qGRAS27-R	(5´-TTCAACTACCATCAACACAAC -3´)<br>(5´- TACCTTCTTGCCAACATATAG -3´)	This work
SlPT4 [Solyc06g051850.1]	qSlPT4-F<br>qSlPT4-R	(5´-GAAGGGGAGCCATTTAATGTGG-3´)<br>(5´-ATCGCGGCTTGTTTAGCATTTCC-3´)	Balestrini *et al.* (2007)
AMT2 [Solyc08g067080.1]	qAMT2-F<br>qAMT2-R	(5´-CTCAGAATGTCAGAGGAAGAT- 3´)<br>(5´-CCAGCAGCAGTATCAGAA-3´)	Ho-Plágaro *et al.* (2021)
RAM2 [Solyc02g087500.1]	qRAM2-F<br>qRAM2-R	(5´-CATGAATCCTAGCCCATC-3´)<br>(5´-GCACTCATACGATAATGTTG-3´)	This work
CCD7 [Solyc01g090660]	qccd7-F<br>qccd7-R	(5´-AGCCAAGAATTCGAGATCCC-3´)<br>(5´-GGAGAAAGCCCACATACTGC-3´)	López-Ráez *et al.* (2010)
SlCyclops [Solyc08g075760.3]	qCyclops-F<br>qCyclops-R	(5´-CAAGGGACATATCAGGAC-3´)<br>(5´-AGGGAGCCATAATACTTTC-3´)	Ho‐Plágaro *et al.* (2021)
SlCCaMK [Solyc01g096820]	qCCaMK-F<br>qCCaMK-R	(5´-GAAGAGGTGTTAAGAGCAATG-3´)<br>(5´-CTCATATCAACCGTTCCATC-3´)	Ho‐Plágaro *et al.* (2021)
SlGRAS38 [Solyc07g052960]	qGRAS38-F<br>qGRAS38-R	(5´-TTGGGAGCCTTGATGGAGC-3´)<br>(5´-GCCCTAAGGAACCATGATGTGA-3´)	This work
SlDLK2 [Solyc05g018413]	qDLK2-F<br>qDLK2-R	(5´-GGGAGTTGAAATTGCATTACCT-3´)<br>(5´-TAGTGAAATGGGCACCACAA-3´)	García Garrido *et al.* (2010)
SlMAX1 [Solyc08g062950]	qMAX1-F<br>qMAX1-R	(5´-CGCCCTTAGTTGCCAGAGAA-3´)<br>(5´-GCCAACCAAACCCATGTTCC-3´)	Guillotin *et al.* (2017)
SlGH3.4 [Solyc02g092820]	qGH3.4-F<br>qGH3.4-R	(5´-CTCCAGGGTGATTTCTGT-3´)<br>(5´-TTCTTTGGTCCACTGTCT-3´)	Liao *et al.* (2015)
SlTPSI1 [Solyc02g092820]	qTPSI1-F<br>qTPSI1-R	(5´-GAGGTGGCTCTCGTCGTTGAT-3´)<br>(5´-TCTGCCTTATCCTTGAGATTGC-3´)	Kohlen *et al.* (2012)

Table S2. MRM conditions for endogenous strigolactones and GR24.

Compound	MRM (Colision energy eV)		
	Quantifier	Qualifier 1	Qualifier 2
Solanacol	343.00 > 183.20 (-21)	343.00 > 96.90 (-11)	343.00 > 240.30 (-30)
5-Deoxystrigol	353.00 > 256.10 (-14)	353.00 > 241.10 (-24)	353.00 > 235.05 (-12)
Fabacyl acetate	405.10 > 97.00 (-30)	405.10 > 345.20 (-15)	405.10 > 231.00 (-20)
Orobanchol	347.10 > 233.15 (-12)	347.10 > 96.90 (-22)	347.10 > 205.20 (-18)
Orobanchyl acetate	411.00 > 254.00 (-15)	411.00 > 239.00 (-20)	-
Sorgolactone	339.20 > 242.20 (-14)	339.20 > 227.05 (-22)	-
Strigol	369.20 > 271.75 (-20)	369.20 > 257.15 (-24)	369.20 > 351.00 (-13)
GR24	299.00 > 281.10 (-7)	299.00 > 97.00 (-22)	299.00 > 185.05 (-9)

Table S3. Number of mapped reads, high quality reads and splices reads for libraries from each sample in the RNA-seq analysis.

Sample name	Total reads	Mapped reads		HQ reads		Splice reads	
		Number	%	Number	%	Number	%
**NI (1)**	78377426	75071296	95.78	53572512	68.35	15320524	19.55
**NI (2)**	58193034	56158555	96.5	35442090	60.9	9980184	17.15
**NI (3)**	62613798	60239532	96.21	39973168	63.84	11900575	19.01
***UBIL:SlRAM1*-NI (1)**	69147934	66935435	96.8	35731200	51.67	10642395	15.39
***UBIL:SlRAM1*-NI (2)**	60380858	58308462	96.57	35162698	58.23	10401885	17.23
***UBIL:SlRAM1*-NI (3)**	58345966	54173739	92.85	43822152	75.11	13007512	22.29
**Average ± SE**	64.51 million ± 3.23	61.81 million ± 3.20	95.79 % ± 0.60	40.62 million ± 2.94	63.02 % ± 3.32	11.88 million ± 0.83	18.44 % ± 0.98

Table S4 List of DEGs genes. List of DEGs generated by RNA-seq found to be differentially expressed upon *UBUL:SlRAM1* expression in non-inoculated roots and by AM inoculation (Displayed as a separate excel file).

Table S5 Gene ontology and protein class analyses on *UBIL:SlRAM1* induced and repressed genes. Selection of enriched slim GO terms and protein class annotations obtained by submitting to the PANTHER database the 1232 genes significantly upregulated (Fold change >2; *P*<0.05) and the 2129 genes significantly repressed (Fold change <-2; P<0.05) by *UBIL:SlRAM1* in our RNA-seq analyses. Its overrepresentation in the AM-induced or AM-repressed genes datasets (Fold change >2 or <-2; *P*<0.05; NCBI Bioproject PRJNA509606) is also indicated.

		***SlRAM1* OE induced**		***SlRAM1* OE repressed**		***AM* induced**		***AM* repressed**	
		fold enrichment	FDR	fold enrichment	FDR	fold enrichment	FDR	fold enrichment	FDR
**Biological Process**	amino sugar catabolic process	24.68	4.87E-02			14.52	1.86E-02		
	organic acid catabolic process	5.11	3.41E-04			2.40	3.04E-02		
	response to wounding			11.60	8.84E-05	5.45	3.06E-02		
	regulation of defense response			11.60	7.37E-05	5.45	3.03E-02		
	regulation of response to stress			6.87	9.61E-04	3.98	4.16E-02		
**Molecular Function**	chitinase activity	10.47	1.68E-05			4.62	2.11E-02		
	hormone binding	6.86	1.46E-03			4.24	2.78E-02		
	inorganic anion transmembrane transporter activity	6.63	5.08E-04			4.15	2.01E-02		
	organic hydroxy compound metabolic process			2.20	3.35E-02	3.35	1.15E-06		
	lipid metabolic process			1.88	5.86E-03	2.67	1.88E-09		
	cellular response to lipid			3.77	5.89E-03	3.69	2.31E-03		
	cellular response to organic substance			2.77	1.85E-03	2.17	2.52E-02		
	cellular response to oxygen-containing compound			2.94	1.90E-02	3.19	2.15E-03		
**Cellular Component**	cell wall	3.92	4.44E-03			6.00	5.79E-16		
	extracellular region	3.34	9.57E-03			2.27	1.91E-02		
	plant-type vacuole membrane			3.71	2.05E-02	3.55	8.58E-03		
**Protein Class**	peroxidase	6.07	1.16E-07			6.20	7.20E-17		
	protease inhibitor	5.56	7.68E-05			5.11	1.48E-08		
	phosphatase	3.80	6.38E-05			2.42	1.16E-03		
	hydrolase			2.10	5.59E-09	2.26	1.53E-14		
	helix-turn-helix transcription factor			1.88	4.64E-02			3.14	3.11E-02
	lipase			2.59	2.60E-03	2.46	1.42E-03		

References

Balestrini R, Gómez-Ariza J, Lanfranco L, Bonfante P. 2007. Laser microdissection reveals that transcripts for five plant and one fungal phosphate transporter genes are contemporaneously present in arbusculated cells. Molecular Plant-Microbe Interactions **20**, 1055-1062.

Benabdellah K, Merlos MA, Azcon-Aguilar C, Ferrol N. 2009. GintGRX1, the first characterized glomeromycotan glutaredoxin, is a multifunctional enzyme that responds to oxidative stress. Fungal Genetics and Biology **46**, 94-103.

García Garrido JM, León Morcillo RJ, Martín Rodríguez JA, Ocampo Bote JA. 2010. Variations in the mycorrhization characteristics in roots of wild-type and ABA-deficient tomato are accompanied by specific transcriptomic alterations. Molecular Plant-Microbe Interactions **23**, 651-664.

Guillotin B, Etemadi M, Audran C, Bouzayen M, Bécard G, Combier JP. 2017. *Sl-IAA27* regulates strigolactone biosynthesis and mycorrhization in tomato (var. MicroTom). New Phytologist **213**, 1124-1132.

Ho-Plágaro T, Morcillo RJ, Tamayo-Navarrete MI, Huertas R, Molinero-Rosales N, López-Ráez JA, Macho AP, García-Garrido JM. 2021. DLK2 regulates arbuscule hyphal branching during arbuscular mycorrhizal symbiosis. New Phytologist **229**, 548-562.

Kohlen W, Charnikhova T, Lammers M, Pollina T, Tóth P, Haider I, Pozo MJ, de Maagd RA, Ruyter-Spira C, Bouwmeester HJ. 2012. The tomato *CAROTENOID CLEAVAGE DIOXYGENASE 8* (*SlCCD8*) regulates rhizosphere signaling, plant architecture and affects reproductive development through strigolactone biosynthesis. New Phytologist **196**, 535-547.

Liao D, Chen X, Chen A, Wang H, Liu J, Liu J, Gu M, Sun S, Xu G. 2015. The characterization of six auxin-induced tomato *GH3* genes uncovers a member, *SlGH3. 4*, strongly responsive to arbuscular mycorrhizal symbiosis. Plant and Cell Physiology **56**, 674-687.

Liu C, Muchhal US, Raghothama K. 1997. Differential expression of *TPSI1*, a phosphate starvation-induced gene in tomato. Plant Molecular Biology **33**, 867-874.

López-Ráez JA, Verhage A, Fernandez I, Garcia JM, Azcón-Aguilar C, Flors V, Pozo MJ. 2010. Hormonal and transcriptional profiles highlight common and differential host responses to arbuscular mycorrhizal fungi and the regulation of the oxylipin pathway. Journal of Experimental Botany **61**, 2589-2601.
